# Molecular Theory of Detonation Initiation: Insight from First Principles Modeling of the Decomposition Mechanisms of Organic Nitro Energetic Materials

**DOI:** 10.3390/molecules21020236

**Published:** 2016-02-19

**Authors:** Roman V. Tsyshevsky, Onise Sharia, Maija M. Kuklja

**Affiliations:** MSE Department, University of Maryland, College Park, MD 20742, USA; rtsyshev@umd.edu (R.V.T.); osharia@gmail.com (O.S.)

**Keywords:** sensitivity, PETN, HMX, TATB, DADNE, BNFF

## Abstract

This review presents a concept, which assumes that thermal decomposition processes play a major role in defining the sensitivity of organic energetic materials to detonation initiation. As a science and engineering community we are still far away from having a comprehensive molecular detonation initiation theory in a widely agreed upon form. However, recent advances in experimental and theoretical methods allow for a constructive and rigorous approach to design and test the theory or at least some of its fundamental building blocks. In this review, we analyzed a set of select experimental and theoretical articles, which were augmented by our own first principles modeling and simulations, to reveal new trends in energetic materials and to refine known existing correlations between their structures, properties, and functions. Our consideration is intentionally limited to the processes of thermally stimulated chemical reactions at the earliest stage of decomposition of molecules and materials containing defects.

## 1. Introduction

While detonation phenomena [1,2,3,4,5,6] were first observed a long time ago, a consistent microscale theory of detonation initiation does not yet exist [7,8]. Despite the relative maturity of the fields of shock wave physics [9,10] and chemistry of materials [11,12], detailed knowledge of the factors that govern the sensitivity of materials to the initiation of chemistry triggered by an external perturbation [13,14,15,16] has yet to be obtained, and fundamental understanding of underlying processes have yet to be established. These uncertainties hamper both the development of novel materials with tailored properties and advances of novel technologies to improve quality of life in modern society.

To be useful, high energy density materials, which possess a lot of energy that can be released upon mild external stimuli, should meet specific requirements, such as ease of synthesis, cost-efficiency, high performance, stability, and reliable safety during handling and transportation. Among the most important, most challenging, and probably least understood criteria is the low sensitivity of energetic materials to detonation initiation, dictating that the explosive decomposition chemistry will be initiated only on demand and not accidently in response to unintended perturbation.

The initiation of detonation is a complex, multi-scale process. The persistent mystery surrounding this process has inspired an exhaustive search for a primary parameter that would elucidate the intricacies of initiation sensitivity. The most notable among these attempts establish a connection between the material’s sensitivity and its electronic structure [17], the band gap dynamics [18,19,20,21], the energies of relevant electronic excitations [22,23], phonon interactions [24], the energy dissipation due to plastic deformation [25], the molecular crystal packing efficiency [26,27,28,29], and intermolecular interactions in the crystal [30,31,32]. Other efforts refer to correlations between the material’s sensitivity and the properties of isolated molecules that comprise it; for example, the electronic charge distribution [33,34,35,36], the electronegativity [37], a combination of the quantum chemical descriptors (energies of molecular orbitals, dipole moments, and ionization potentials) [38], and selected structural parameters [39,40,41,42].

Some researchers have posited that the impact sensitivity of high explosive materials depends on the thermal stability of the molecules [43,44] rate constants [45], acid-base interactions, [46] and the oxygen balance [47]. Another set of studies employed an esoteric methodology of artificial neural networks [48,49] to link the material’s sensitivity to a combination of molecular properties (the number of atoms and functional groups, bond lengths and bond angles, HOMO-LUMO energies and ionization potentials, heats of formation and bond dissociation enthalpies) [48,50].

These examples represent a fraction of publications devoted to discovering a direct correlation between sensitivity and a single descriptive parameter. It is evident, however, that a problem of such complexity requires a more elegant solution.

In this concept review, we intentionally limited our consideration to the processes of thermally stimulated chemical reactions at the earliest stage of decomposition of molecules and realistic materials containing defects. While a thermal contribution may accompany other perturbations, such as a mechanical impact or electrical shock, it is prudent to separate different effects of the materials’ response to external excitations for both the simplicity and clarity of description. A notion that thermal decomposition processes play a major role in defining the sensitivity of organic energetic materials to detonation initiation was sketched in a recent review [51]. In this paper, we systematize trends in energetic materials, analyze newly suggested chemical mechanisms, and refine existing known correlations between their structures, properties, and functions. Our overarching goal is to initiate a rigorous analytic (rather than empirical) formulation of molecular initiation of detonation theory.

**Figure 1:**
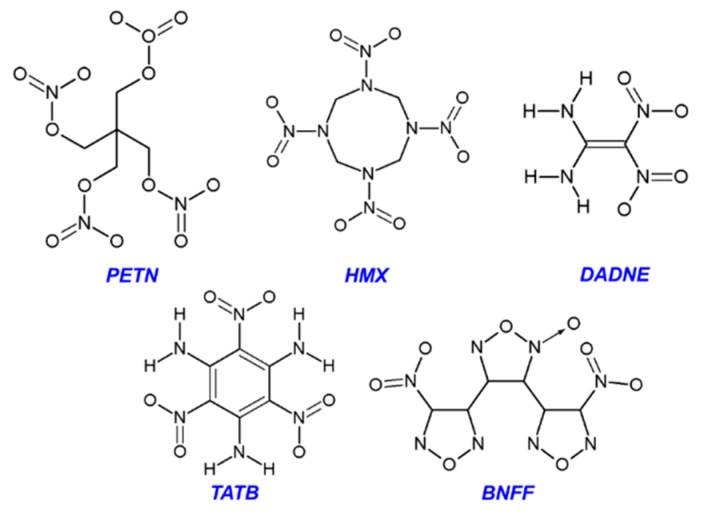
Schematic of PETN, HMX, DADNE, TATB, and BNFF molecular structures.

Here we focused on an atomistic first principles analysis of initial decomposition reactions in five classes of nitro energetic crystals, with a general formula of C_a_H_b_N_c_O_d_, we address several fundamental issues: (i) the molecular properties, while providing a useful insight into material’s chemistry, are insufficient due to the lack of intermolecular interactions; (ii) the realistic simulations should take into account the morphology of crystals and defects; and (iii) the initiation chemistry is defined by the interplay of co-existing reactions that strongly depend upon the chemical composition of the material and its structural arrangement. We report here an analytical review to compare two traditional high explosives, nitro ester pentaerythritol tetranitrate (PETN, C_5_H_8_N_4_O_12_) and nitramine cyclotetramethylene-tetranitramine (HMX, C_4_H_8_N_8_O_8_) with C-nitro compounds, triamino-trinitrobenzene (TATB, C_6_H_6_N_6_O_6_) and experimental explosive diamino-dinitroethene (DADNE, C_2_H_4_N_4_O_4_), which recently gained popularity, and bis-(nitrofurazano)-furoxane (BNFF, C_6_N_8_O_8_), a novel promising heterocyclic energetic material (Figure 1). A large set of experimental and theoretical data found in literature is augmented by our own calculations of chemical reactions in the organic molecules and the corresponding materials containing defects. We used only select, most relevant published articles for the analysis and illustrations and hence some papers, which are also relevant to this review, were not included in our consideration.

## 2. Computational Details

Our quantum-chemical calculations were performed using the approach [51,52,53,54,55] that links density functional theory (DFT) [56,57], variational transition state theory (TST), and *ab initio* chemical kinetics with a realistic description of molecules and defect containing crystals.

### 2.1. Gas-Phase Calculations

Modeling of gas-phase reactions is an essential and necessary part in all studies of thermally induced decomposition of nitro compounds. Modern algorithms and methods implemented in software packages allow us to obtain accurate data on geometry and vibrational structures of reagents, intermediates, products, and transitions states, model reaction pathways, calculate activation barriers, reaction energies, and pre-exponential factors. These factors provide a solid background for discussion of the competition between chemical reaction mechanisms.

Chemical reactions in the gas-phase were modeled using DFT in the GGA approximation with the PBE functional [58] and refined with hybrid exchange correlation-corrected PBE0 functional [59]. In our calculations, we use a set of available hybrid functionals depending on a specific problem. For example, fragmentation pathways of TATB and BNFF molecules were additionally explored using B3LYP, which includes Becke’s three parameter [60] and the Lee–Yang–Parr correlation [61] functionals. All gas-phase calculations were performed using the split-valence double-zeta 6-31+G(2df,p) basis set as implemented in GAUSSIAN09 code [62]. Vibrational frequencies were calculated for relevant atomistic configurations to distinguish energy minima and transition states and to determine corresponding zero-point energy (ZPE) corrections. The stationary points corresponding to the energy minimum were positively identified by having no imaginary frequencies, and the transition states were confirmed to have exactly one imaginary frequency. An intrinsic reaction coordinate analysis was carried out by using the Hessian-based Predictor-Corrector integrator algorithm [63,64] for each transition state.

### 2.2. Periodic Calculations

Periodic solid-state calculations were performed using the GGA PBE functional and PAW pseudo-potentials [65] as implemented in the plane wave VASP code [66,67,68]. Using an ideal bulk structure of each molecular crystal under study, test calculations for energy convergence as a function of the number of *k*-points and the kinetic energy cut-off are required. In the smallest supercells that are needed for simulations of chemical reactions, we used 2 × 2 × 2 Monkhorst−Pack *k*-point mesh in modeling ideal bulk structures of crystals (e.g., PETN, β-HMX, and DADNE). The kinetic energy cut-offs range from 520 to 800 eV, depending on the material. More details of DADNE and TATB calculations are provided in Supplementary Materials.

Full relaxation of ionic positions and lattice parameters of ideal bulk structures, initially taken from experiment, was carried out using conjugate-gradient algorithm. The convergence criterion for electronic steps was set to 10^−5^ eV, and the maximum force acting on any atom was set not to exceed 0.02 eV/Å. The lattice parameters of the crystalline structures calculated using PBE level were in reasonable agreement with experiment (1%–3%) [51], which is within typical GGA accuracy.

Modeling of chemical reactions of molecular crystals containing defects (including surfaces, vacancies, voids, or dislocations) requires constructing large supercells composed out of several hundred atoms (~500–1000). The large size of supercells serves to avoid unphysical interactions between reacting molecules (products or defects) in periodically repeated cells. Surface reactions are simulated in a slab model, in which the supercell consisted of a crystalline layer cut out of a molecular crystal in a certain direction. A 10–15 Å-thick vacuum layer, placed on top of the slab, also serves to eliminate artificial interactions between reaction products and surface fragments in directions normal to the surface plane. Whenever possible, we use in calculations surface structures that correspond to low-energy facets of crystals observed in experiment [69,70]. For newly designed or predicted materials, we study surfaces, which are likely to have a low energy based on a simplified theoretical analysis of their crystalline structures, symmetry, and possible slip systems.

Minimal energy paths in VASP periodic calculations were obtained with the nudged elastic band method [71]. Pre-exponential factors were calculated with conventional transition state theory [72] for the decomposition reactions that proceed through a formation of a transition state and with variational transition state theory [73] for the homolytic cleavage pathways. 

The underestimated activation barriers, the trend characteristic of standard DFT functionals [74,75], were refined by running single point energy calculations using the hybrid PBE0 and B3LYP functionals based on the structures of reagents and transition states obtained with PBE. This technique is successfully employed to study the decomposition kinetics of nitro compounds in solid state.

## 3. Results and Discussion

We analyze multiple reaction mechanisms along with their kinetics and compare various aspects of the thermal decomposition processes to reveal similarities and differences between them. These comparisons include (a) activation barriers and reaction rates of competing reactions; (b) several classes of nitro compounds; (c) molecules and crystals; (d) ideal crystals and materials containing defects; and (e) functional groups and their structural placements in the molecule. There is an important difference in chemical bonding of nitro groups to the core of the molecule. In nitrate esters, NO_2_ is connected to an oxygen atom (R–O–NO_2_), in nitramines, —to a nitrogen atom (R–N–NO_2_), and in C-nitro explosives, —to a carbon atom (R–C–NO_2_). This feature is often called a critical bond the increasing strength of which is correlated with the decreasing thermal sensitivity in a series PETN > HMX > DADNE and typically attributed to the lower activation barrier of the NO_2_ loss in PETN than in HMX and DADNE [43,51].

### 3.1. PETN

**Figure 2:**
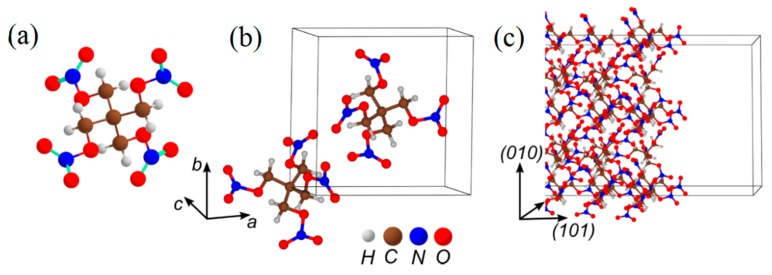
The structure of (**a**) the PETN molecule; (**b**) the PETN crystal; and (**c**) the model supercell representing the (101) PETN surface.

Nitrate ester PETN (Figure 2) closely related to nitroglycerin is known for its dual use as a medication and a high explosive. The most stable of the nitrate esters, PETN is the most sensitive secondary high explosive in its class, and hence it is often called a benchmark compound [76,77] that classifies all materials, with the sensitivity higher than PETN, as primary explosives. 

Commonly registered products of PETN decomposition are CO, CO_2_, NO, N_2_O, CH_2_O, HCN, HNCO, and most researchers agree that the initial decomposition step is the O-NO_2_ bond homolysis [78]. Reported experimental activation energies, *E*_a_, of PETN decomposition are largely scattered, ranging from 30 to 70 kcal/mol [79,80,81,82,83,84,85,86]. Potential ground state decomposition mechanisms of PETN were recently scrupulously explored [87]. To avoid repetitions, we will include only two mechanisms that are most important for the initiation stage (Equations (1) and (2)).

**Figure 3:**
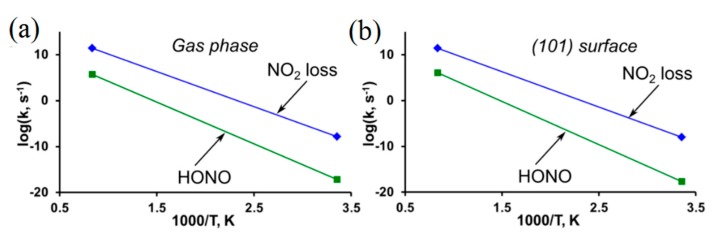
Reaction rates are displayed for the decomposition of PETN (**a**) in the gas phase and (**b**) on the (101) surface.

The homolytic cleavage of the O-NO_2_ bond (Equation (1)) is found to be the most kinetically and energetically favorable decomposition channel of the PETN molecule [88] and the (101) and (110) PETN surfaces [87]. The calculated BDE (~35 kcal/mol, Table 1) is in good agreement with the differential scanning calorimetry (DSC) measurement of 32.6 kcal/mol [86] and with a range of earlier experimental studies, 35.0 [85]–39.5 [82,89]. The elimination of the nitrous acid (HONO) (Equation (2)) is also considered a possible decomposition channel for nitro compounds containing a hydrogen atom in the β-position relative to a nitro group. This mechanism requires a slightly higher (~41 kcal/mol, Table 1) energy than the O-NO_2_ bond scission, though the obtained activation barrier falls in the experimental range and is in general agreement with other theoretical results, 36–39 kcal/mol [88]. The HONO elimination is a much slower reaction than the NO_2_ loss (Figure 3a,b) due to the low pre-exponential factor (~13, Table 1).

C_a_H_b_N_c_O_d_ → C_a_H_b_N_c-1_O_d-2_^●^ + ^●^NO_2_ (1)

C_a_H_b_N_c_O_d_ → C_a_H_b-1_N_c-1_O_d-2_ + HONO (2)

Extrapolating these findings to other nitrate esters, we can draw an important conclusion that the earliest stages of the initiation chemistry are defined by the relationship of the fast endothermic NO_2_ loss reaction and the slow exothermic HONO elimination reaction. Note, the activation barrier for the molecular gas-phase decomposition (~35 kcal/mol) almost coincides with that of the (101) surface decomposition (Table 1).

### 3.2. β-HMX

**Figure 4:**
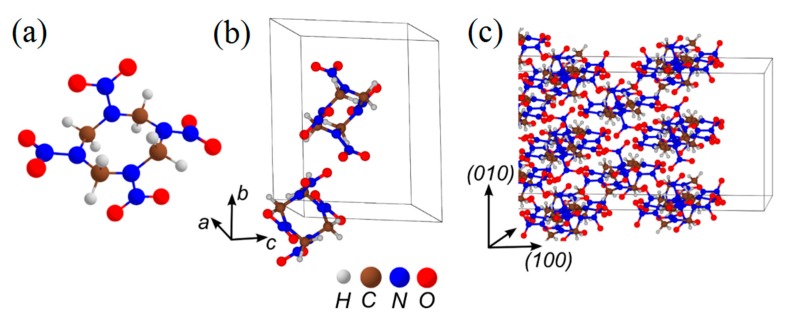
The structure of (**a**) an HMX molecule; (**b**) an HMX ideal crystal; and (**c**) a model supercell fragment of the (100) HMX surface.

Nitramine HMX (C_4_H_8_N_8_O_8_, Figure 4a–c) [90], closely relevant to cyclotrimethylene-trinitramine (RDX, C_3_H_4_N_4_O_4_), is a conventional, widely used explosive and serves as a vivid representative of the nitramine class of energetics.

The main products of thermal decomposition of HMX at ambient and low pressure are CH_2_O, N_2_O, HCN, HONO and NO_2_. As the heating rate and temperature increase, HCN and NO_2_ dominate and CH_2_O and N_2_O play a lesser role [91]. The DSC and TGA measurements [92] of the HMX decomposition suggested that the activation barrier, *E*_a_, falls in the range from 33.7 to 46.6 kcal/mol and the estimated pre-exponential factor log *A* is between 11 and 17 (s^−1^). A logically justified and data-supported explanation to the previously reported unusually wide range of data (from 13 to 67 kcal/mol) [93,94] was developed only recently [52,53,95,96].

**Figure 5:**
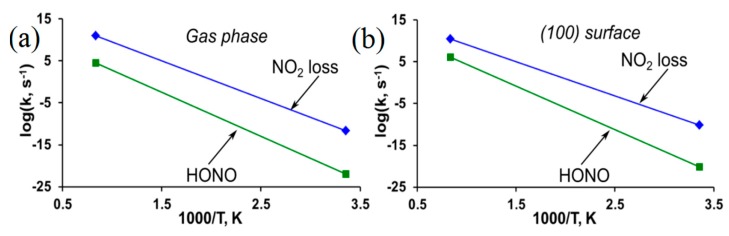
Reaction rates are displayed for the decomposition of β-HMX (**a**) in the gas phase and (**b**) on the (100) surface.

Similarly to nitrate esters, the homolytic NO_2_ loss (Equation (1)) is the most favorable pathway of the thermal decomposition of β-HMX in the gaseous phase [97] and solid state [53,55] (Figure 5a,b). The calculated energy of the N-NO_2_ homolysis of the isolated molecule (~41 kcal/mol, Table 1) falls in the interval of 32.1 to 52.9 kcal/mol reported for the gaseous phase [93], and is in reasonable agreement with the experimental activation energies of 38.0 [98], 39.6 [99] and 46.2 [100] kcal/mol. The cleavage of N-NO_2_ in the HMX molecule placed on the (100) surface requires slightly lower (~37 kcal/mol, Table 1) energy than the bond dissociation of the isolated molecule (~41 kcal/mol). The activation barrier of the HONO elimination from β-HMX (~47 kcal/mol) is higher for both the gas and the surface decompositions than the corresponding cases of the N-NO_2_ homolysis. Notably, the HONO elimination exhibits the similar barriers in the molecule and the surfaces, meaning that this is an essentially intramolecular dissociation mechanism, which is barely affected by intermolecular interactions. In addition, the HONO is a noticeably slower reaction than the N-NO_2_ homolysis in both gaseous phase and on the (100) surface (Figure 5a,b) due to the lower pre-exponential factor and higher activation barrier (Table 1).

### 3.3. δ-HMX

**Figure 6:**
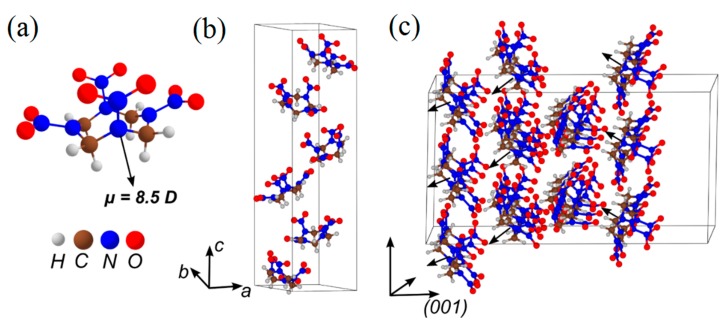
The structure of (**a**) a boat conformer of δ-HMX molecule with the dipole moment μ; (**b**) an δ-HMX ideal crystal; and (**c**) a model supercell fragment of the (001) δ-HMX surface.

The δ-HMX-based materials are widely used as solid fuels for rocket and space ship engines. In laboratories, δ-HMX crystals [101] (Figure 6a–c) and composites are studied due to their significantly higher sensitivity and higher burn rates than those of β-HMX [102]. The δ-HMX phase crystals are usually obtained from β-HMX phase samples by heating them above 160 °C and stimulating the β→δ phase transition [103,104,105]. In attempts to explain the differences in reactivity of two phases, the high sensitivity of the δ-phase (in comparison to the β-phase) was attributed to a growing number of voids during the phase transformation [106,107,108,109] and changes of the molecular structure [110,111] of the material.

Experimental measurements of δ-HMX thermal decomposition are largely lacking with an exception of two studies [112,113] that reported the activation barrier of 34.4 kcal/mol and the pre-exponential factor of log (*A*, s^−1^) = 15.3.

A comparative analysis of thermal decomposition reactions of the δ-HMX molecule (boat conformation, Figure 6a) and the β-HMX (chair conformation, Figure 4a) indicated no difference in dissociation mechanisms and kinetics (Table 1) [53,54,55,95,96]. Thus, the changes of the molecular structure are ruled out as a possible reason for differences in sensitivities. The homolytic cleavage of the N-NO_2_ bond Equation (1) remains the predominant decomposition pathway in both phases and requires similar energies, 41.5 kcal/mol in δ-HMX and 40.9 kcal/mol in β-HMX (Table 1). Also in both δ-HMX and β-HMX, the HONO elimination Equation (2), will unlikely compete with the NO_2_ loss due to a high activation barrier (44.1 kcal/mol, Table 1) and a low pre-exponential factor (log(*A*, s^−1^) = 13.3, Table 1).

Only simulations of HMX containing defects (ion radicals, charge transfer, and HMX surfaces) made an important reveal. It was shown that the polar (001) δ-HMX surface decomposes quite differently than any nonpolar β-HMX surface. An electric field, generated by the molecular dipoles, induces a charge transfer to form positively and negatively charged surfaces (Figure 6c). Such a charge separation creates favorable conditions for initiation of a fast reaction [51,96] thus imposing a crucial effect on thermal stability of δ-HMX. The β-HMX crystals do not have any polar surfaces and hence they do not suffer from polarization-induced instability.

**Figure 7:**
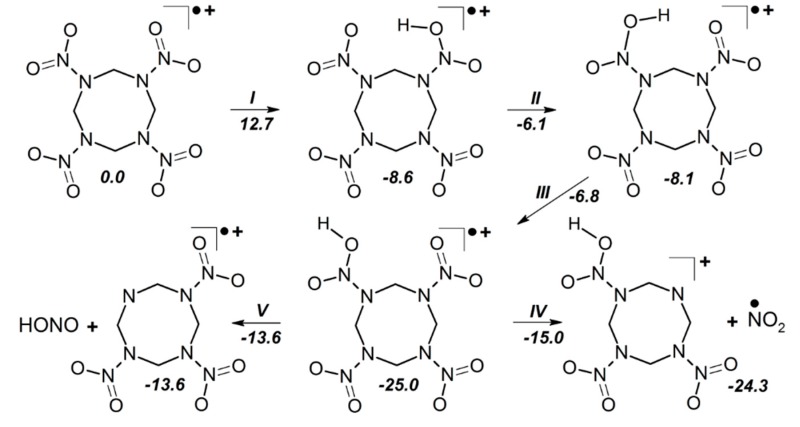
A schematic representation of fragmentation pathways of δ-HMX radical cation.

Speaking in terms of activation barriers, the decomposition of δ-HMX on the positively charged surface reminds the decomposition of a radical cation (Figure 7). The NO_2_ loss and HONO elimination on the positively charged δ-HMX surface requires 20.7 kcal/mol and proceeds via the δ-HMX *aci*-isomer formation (*I*, Figure 7). The N-NO_2_ homolysis on the negatively charged surface requires 20.1 kcal/mol (Equation (3), Table 1) and mimics the process in the δ-HMX radical anion (17.0 kcal/mol, Table 1):

C_4_H_8_N_8_O_8_^●^**^−^**→ C_4_H_8_N_7_O_6_**^−^** + ^●^NO_2_ (3)

Interestingly, the obtained activation barriers for the δ-HMX surfaces (and also for ion radicals) are dramatically lower that these for the corresponding reactions on nonpolar β-HMX surfaces. It is tempting to associate these low energies and fast kinetics with rather high sensitivity of δ-HMX to thermal initiation. Two important implications immediately follow from this knowledge. The activation barriers and hence the sensitivity of polar δ-HMX are comparable to those of primary explosives, metal azides, SrN_6_ (20 kcal/mol), CuN_3_ (27 kcal/mol), PbN_6_ (29 kcal/mol) and AgN_3_ (32 kcal/mol) [114]. Further, the wide range of experimental activation barriers reported for HMX can be attributed to various crystallographic phases, including both β- and δ-HMX. With polar δ-HMX facets requiring appreciably lower activation energy than β-HMX facets, an unusually large scattering of activation barriers in the overall thermal initiation should be expected.

### 3.4. DADNE

**Figure 8:**
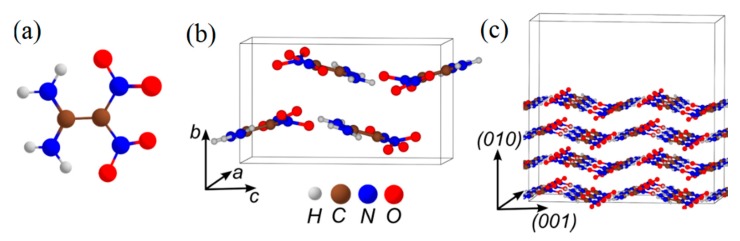
The structure of (**a**) a DADNE molecule; (**b**) a DADNE ideal crystal; and (**c**) a model supercell fragment of the (010) surface.

Details of synthesis [115], structure [116] and physico-chemical properties [117] of DADNE (Figure 8) were reported in 1998 when the material had a promise to combine two important properties, high performance and low sensitivity. Structurally, DADNE (C_2_H_4_N_4_O_4_) is often compared to HMX (C_4_H_8_N_8_O_8_), RDX (C_3_H_6_N_6_O_6_), and TATB (C_6_H_6_N_6_O_6_). The high performance of DADNE, similar to HMX and RDX was attributed to the same stoichiometry (the DADNE molecule represents ½ of an HMX molecule and 2/3 of an RDX molecule). On the other hand, the critical chemical bond of DADNE, C-NO_2_, reminding TATB, inspired high hopes for low sensitivity, analogous to TATB. The molecular configuration of DADNE (Figure 8a) can be described as a push-pull ethylene with two donor amino groups (“head”) and two withdrawing nitro groups (“tail”). In the crystal (Figure 8b), polar DADNE molecules are packed “head-to-tail” forming wave-shaped layers with linear intermolecular hydrogen bonding within the layers and weak van der Waals interactions between the layers [116].

Earlier results of the drop hammer test (h_50%_ = 126–159 cm) pointed toward significantly lower impact sensitivity of DADNE relative to nitramine RDX (h_50%_ = 38 cm) [118]. The reported activation energy of the thermal decomposition of DADNE (58 kcal/mol) [117] is closer to nitroarenes (>60 kcal/mol) than nitroesters (~35 kcal/mol) or nitamines (~40–45 kcal/mol), which also points towards higher thermal stability of the material. Hence, it is not surprising that DADNE became an object of many experimental and theoretical studies. Despite the extensive effort of researchers, many questions regarding stability of DADNE remain unanswered.

Experimental measurements of the activation energy for DADNE decomposition that became available over last two decades appear scattered in the range of 45–80 kcal/mol [117,119,120]. The CO_2_, HCN, N_2_O, NO_2_, HOCN, H_2_O, and NO species were registered among decomposition products of DADNE [117,119].

**Figure 9:**
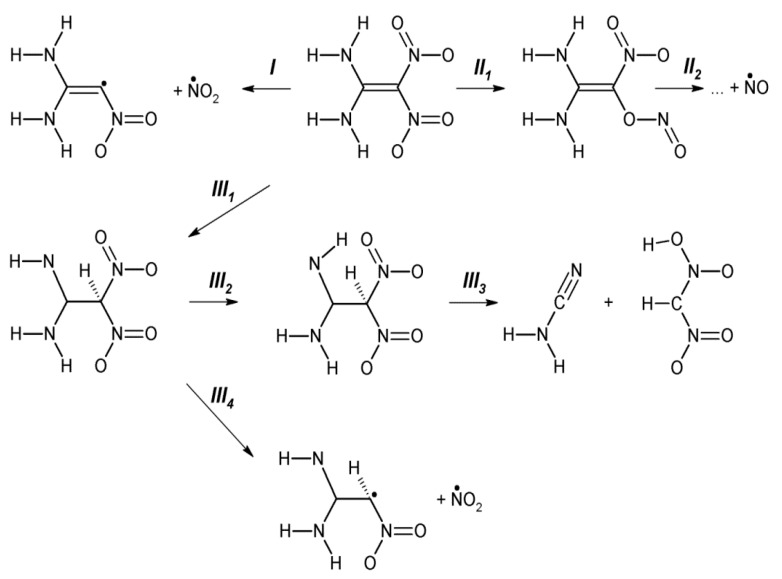
A schematic representation of DADNE fragmentation pathways.

Ordinarily, the C-NO_2_ bond homolysis [121,122] (path *I*, Figure 9) and NO loss proceeding via CONO isomerization [122,123,124] (path *II*, Figure 9) have been considered the main favorable mechanisms of DADNE thermal decomposition. In contrast to a traditional view and earlier conclusions, recent theoretical studies [125,126] proposed yet another possible decomposition mechanism with even lower activation barrier (~45 kcal/mol) proceeding via an enamino-imino isomerization (path *III*, Figure 9) in the gas phase. We rigorously reevaluated conventional reactions and tested newly proposed mechanisms for DADNE decomposition.

Our calculations confirm that the enamine-imine isomerization (path *III*_1_, Figure 9), leading to a formation of 1-amino-1-imino-2,2-dinitroethane (AIDNE), has the lowest activation barrier (49.4 kcal/mol, Table 1) among potential primary decomposition steps in the gas phase, the NO_2_ loss (68.6 kcal/mol) and CONO isomerization (61.5 kcal/mol). There are two possible channels of further decomposition of AIDNE. The first channel involves a rotation of the NH fragment about the C-N bond (path *III*_2_, Figure 9) and the subsequent CH_2_N_2_ elimination via the C-C bond cleavage (path *III*_3_, Figure 9). Both reaction steps *III*_2_ and *III*_3_ (34.4 and 37.4 kcal/mol, Figure 9) require a lower energy than the primary enamine-imine isomerization (Table 1). The second possible channel of AIDNE proceeds via the homolytic C-NO_2_ bond cleavage (path *III*_4_, Figure 9) and requires overall 47.8 kcal/mol (Table 1).

**Figure 10:**
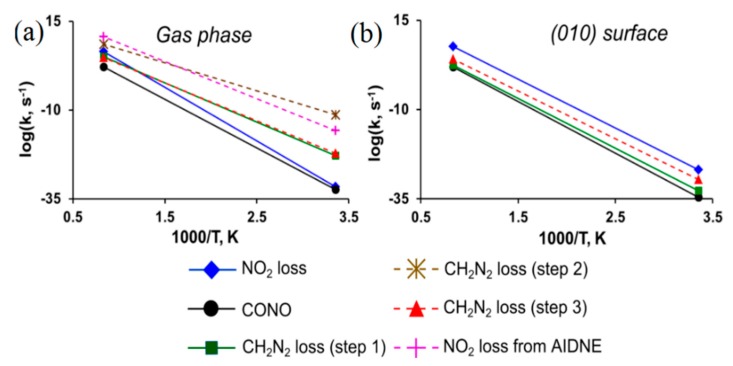
Reaction rates of DADNE decomposition (**a**) in the gas phase and (**b**) on the (010) surface.

The calculated reaction rates (Figure 10a) indicate that the CH_2_N_2_ elimination (path *III*_1_→*III*_3_, Figure 9) and the NO_2_ loss from AIDNE (path *III*_1_→*III*_4_, Figure 9) are predominant decomposition pathways of DADNE in the gas phase, and the primary enamine-imine isomerization (path *III*_1_, Figure 9) should be considered as the rate limiting step.

Further, we simulated the same decomposition mechanisms on DADNE crystals. The homolytic C-NO_2_ bond cleavage (path *I*, Figure 9) proceeding on the (010) surface (Figure 8c) requires slightly lower energy than that in the gas phase, whereas the CONO isomerization (path *II*, Figure 9) on the (010) surface has slightly higher activation barrier than that in the isolated molecule (Table 1). The strongest effect the (010) surface makes on kinetics of fragmentation channels associated with the enamine-imine isomerization (path *III*, Table 1). The activation barriers of the enamine-imine isomerization (62.2 kcal/mol, path *III*_1_, Table 1), rotation of the NH fragment about the C-N bond (60.7 kcal/mol, path *III*_2_, Figure 9) and subsequent CH_2_N_2_ elimination (58.8 kcal/mol, path *III*_3_, Figure 9) are 12–25 kcal/mol higher that the gas-phase reactions (49.4, 34.4 and 47.9 kcal/mol, respectively, Table 1). The NO_2_ loss from AIDNE proceeding on the (010) surface (path *III*_4_, Figure 9) also requires ~16 kcal/mol higher energy (61.9 kcal/mol, Table 1) than in the gas phase (46.2 kcal/mol, Table 1). An increase of activation energies is also reflected in the reaction kinetics. As a result, the enamine-imine isomerization proceeds on the (010) surface (path *III*_1_, Table 1) at lower rates than the NO_2_ loss (Figure 10b). Therefore, we conclude that the homolytic C-NO_2_ bond cleavage remains the predominant decomposition channel of DADNE crystals on the (010) surface.

The thermal decomposition mechanism and kinetics of DADNE are strongly dependent upon the crystal structure. As a result DADNE will likely to decompose in the solid state via the homolytic NO_2_ loss, though several kinetically more favorable fragmentation channels were proposed for gaseous phase. This example with DADNE demonstrates that explicit modeling of the solid state decomposition processes is required for analyzing materials sensitivity and molecular calculations alone do not provide sufficient and reliable information.

### 3.5. TATB

**Figure 11:**
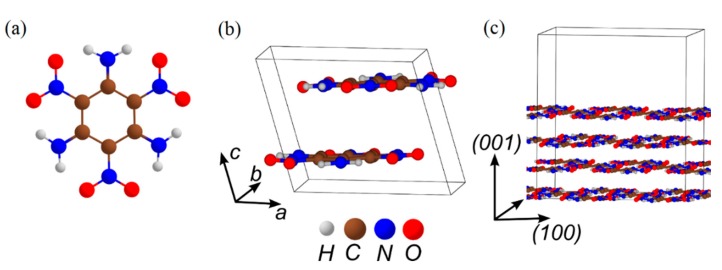
The structure of (**a**) a TATB molecule; (**b**) a TATB ideal crystal; and (**c**) a model supercell fragment of the (001) surface.

TATB (Figure 11) is the benchmark high density energy material that is characterized by the lowest achieved sensitivity against impact (h_50%_ > 320 cm) [127] and against thermal decomposition (E_a_ = 59–68 kcal/mol) [128,129]. There are several theoretical studies reporting the energies of activation barriers for the C-NO_2_ bond scission (64–77 kcal/mol) [44,130,131,132] and CONO isomerization (55 kcal/mol) [124]. Results of some studies also suggest that an elimination of water may be the predominant channel for thermal decomposition of TATB [130]. Surprisingly, however, a detailed theoretical study of thermal decomposition of TATB that analyses both molecular and crystalline processes based on quantum chemical methods is still lacking. Therefore, we simulated several viable fragmentation mechanisms of TATB in the gas phase and solid state aimed at obtaining kinetic data and performing a more comprehensive analysis of thermal sensitivity of this material than was available up to now.

**Figure 12:**
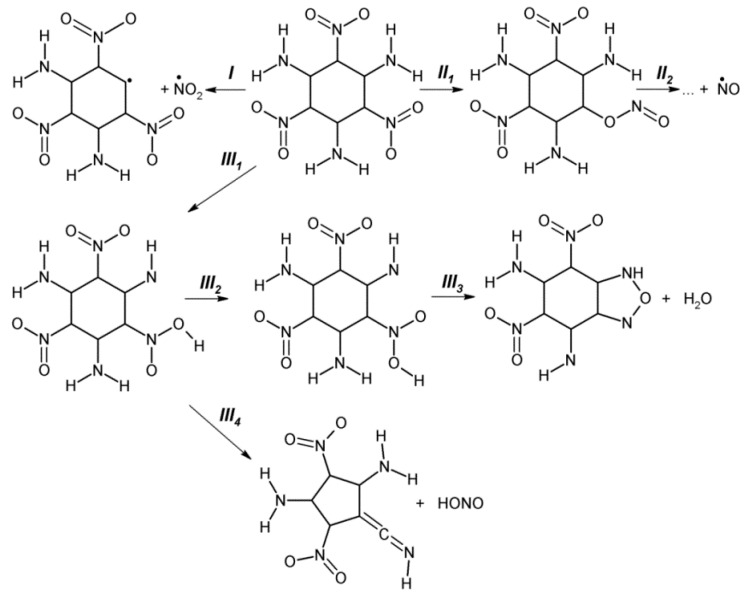
A schematic representation of TATB fragmentation.

We found that the rupture of the C-NO_2_ bond (path *I*, Figure 12) requires 65–70 kcal/mol in the gas phase (Table 1). The CONO isomerization (path *II*, Figure 12) is a less demanding process with the activation barrier of 58.6 kcal/mol (Table 1). An alternative mechanism of water elimination from TATB (path *III*_1_–*III*_3_, Figure 12) is a stepwise process. The primary step (path *III*_1_, Figure 12) involves a formation of the *aci*-isomer of TATB and requires relatively low energy of 42.1 kcal/mol (Table 1). A rotation of the HONO fragment about the C-N bond (path *III*_2_, Figure 12) proceeds during the second step with the overall activation barrier of 68.6 kcal/mol (Table 1). Finally, an elimination of the H_2_O molecule costs 58.8 kcal/mol (Table 1). The stepwise elimination of HONO from TATB (path *III*_1_-*III*_4_, Figure 12) has the highest activation barrier of 94.1 kcal/mol (Table 1).

**Figure 13:**
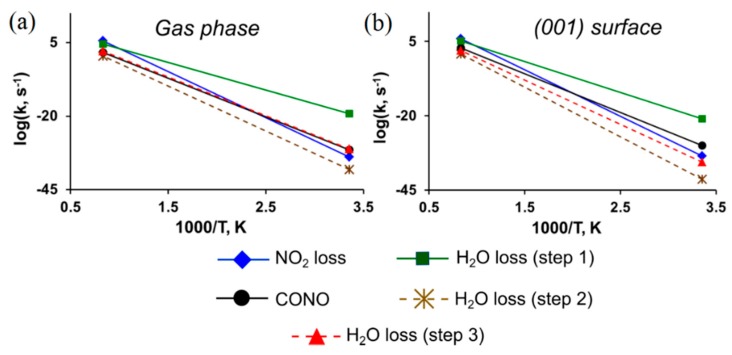
Reaction rates of TATB decomposition (**a**) in the gaseous phase and (**b**) on the (001) surface.

Pre-exponential factors and reaction rates depicted in Figure 13 show that the C-NO_2_ fission is the dominating decomposition pathway in the gas phase at high temperatures (T > 400 K). With the lower activation barrier (by ~8 kcal/mol, Table 1), the CONO isomerization will be the fastest reaction at low temperatures due to relatively low pre-exponential factor (Table 1). Elimination of the water molecule will unlikely compete with these two reactions due to the high overall activation barrier of the rate limiting stage associated with the rotation of the HONO fragment (68.6 kcal/mol, path *III*_2_, Figure 12) and the moderate pre-exponential factor (log(A, s^−1^) = 13.4, Table 1). The HONO loss from TATB (94.1 kcal/mol, path *III*_1_-*III*_4_, Figure 12) is the slowest reaction due to its significantly high activation barrier and relatively low pre-exponential factor (log(A, s^−1^) = 13.4, Table 1). This leads us to exclude this mechanism from further consideration in simulations of thermal decomposition pathways of solid state TATB.

The decomposition of TATB on the (001) surface obeys the same general trends as the process in the gas-phase. All reactions proceeding on the (001) surface have slightly higher activation energies except the CONO isomerization (Table 1). The pre-exponential factors collected in Table 1 and reaction rates depicted in Figure 13 reveal two main competing channels with the NO_2_ loss being the dominating decomposition pathway at T > 500 K and the CONO isomerization dominating at low temperatures. Elimination of water from TATB will unlikely compete with the two channels (Figure 13) due to the high overall activation barriers and moderate pre-exponential factors of secondary reactions (Table 1) associated with the rotation of the HONO fragment in the *aci*-form of TATB (paths *III*_2_, Figure 12) and H_2_O loss (paths *III*_3_, Figure 12). Interestingly, our periodic calculations found that the activation barrier of the primary step, the intramolecular hydrogen transfer, and the formation of *aci*-isomer of TATB (paths *III*_1_, Figure 12), proceeding on (001) surface is 46.1 kcal/mol (Table 1), which is even lower than the earlier estimates of 76.1 kcal/mol [133] obtained using an embedded cluster model. Despite this, the water loss reaction cannot be considered the primary initiation reaction of TATB decomposition.

In general, the calculated here energy of the C-NO_2_ bond scission (65–71 kcal/mol, Table 1) and the activation barrier of the CONO rearrangement (58 kcal/mol, Table 1) are in good agreement with the range of experimentally measured activation energies 59–68 kcal/mol [128,129].

### 3.6. BNFF

**Figure 14:**
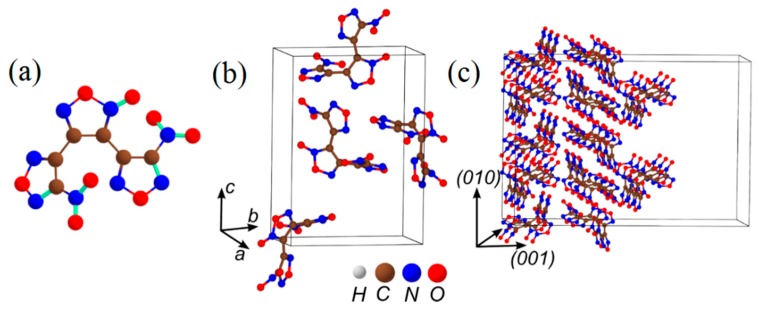
The structure of (**a**) a BNFF molecule; (**b**) a BNFF ideal crystal, and (**c**) a model supercell fragment of the (001) surface.

BNFF (Figure 14) is a recently developed, attractive heterocyclic energetic compound with good thermal stability, low melting point (110 °C), high density (1.937 g/cm^3^), and moderate sensitivity to various insults [134]. BNFF belongs to C-nitro compounds and is predicted to possess a low sensitivity due to the C-NO_2_ bond, heterocyclic molecular structure, and densely packed molecular crystal arrangement (Figure 14a–c). In our study, BNFF represents the whole class of heterocyclic C-nitro explosive materials. The idea here is to explore whether such an arrangement of the molecule and the crystal will gain additional stability against a thermal perturbation and thus improve (*i.e*., lower) the sensitivity of the material to initiation of detonation.

**Figure 15:**
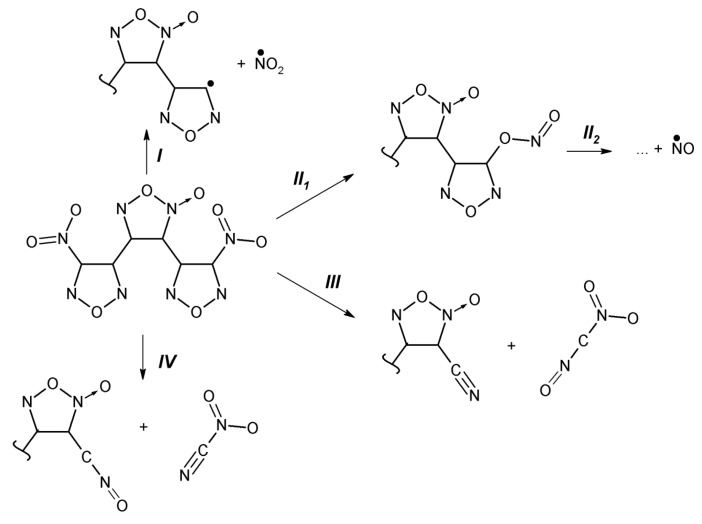
Schematic representation of BNFF fragmentation.

**Figure 16:**
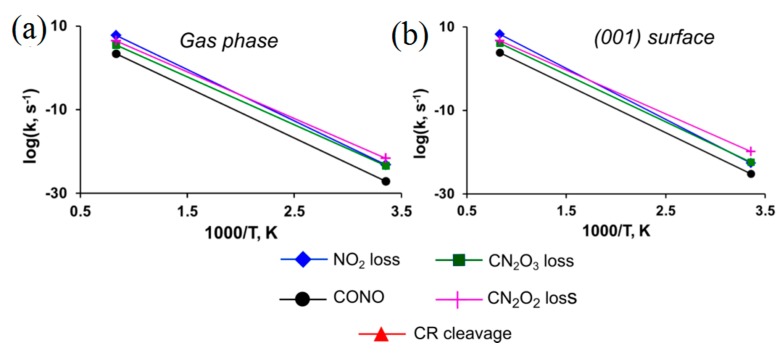
Reaction rates of BNFF decomposition (**a**) in the gas phase and (**b**) on (001) surface.

The calculated energy of the homolytic C-NO_2_ bond cleavage in BNFF (path *I*, Figure 15) is 56–59 kcal/mol (depending on the DFT functional, Table 1), which is comparable with the strength of the C-NO_2_ bond in DADNE and TATB and notably higher than the X-NO_2_ bond fissions in nitrate esters and nitramines (Table 1). The further investigation of alternative mechanisms revealed the reaction pathways, which can be activated with even lower energies than the C-NO_2_ homolysis. For example, the CONO isomerization (54.3 kcal/mol path *II*, Figure 15) requires a somewhat lower activation barrier (by 1–3 kcal/mol, Table 1) than the C-NO_2_ bond fission (56.2 kcal/mol, Table 1), and being an exothermic reaction, releases ~10 kcal/mol of heat (Table 1). However, Figure 16a shows that the CONO isomerization is slower than the NO_2_ loss due to a lower pre-exponential factor (Table 1). The elimination of the CN_2_O_3_ molecule via the outer oxadiazol ring cleavage (path *III*, Figure 15) in BNFF requires 50.5 kcal/mol (Table 1). Another possible pathway for the opening of the outer heterocyclic rings (path *IV*, Figure 15) proceeds via elimination of CN_2_O_2_ and demands even lower energy (48.9 kcal/mol, Table 1) and hence it will dominate during gas-phase decomposition at T < 500 K (Figure 16a).

The elimination of CN_2_O_2_ via concerted breaking of the outer oxadiazol ring remains the predominant decomposition channel on the (001) BNFF surface. The calculated activation barriers of the outer ring cleavage of BNFF on the (001) surface (45.8 kcal/mol, Table 1) seems to agree well with the experimental estimate of 42.3 kcal/mol [135]. The calculated pre-exponential factors (log(A, s^−1^) ~15, Table 1) are also consistent with the experimental value (log(A, s^−1^) = 13.68) [135]. The scission of the C-NO_2_ bond in BNFF on the (001) surface, although requires 3.1 kcal/mol lower energy than that in the gas-phase (Table 1), is still significantly (~10 kcal/mol, Table 1) higher than the activation barrier of the CN_2_O_2_ loss. As the result, the CN_2_O_2_ elimination will dominate the decomposition on the (001) surface at T < 650 K (Figure 16b), whereas the C-NO_2_ loss will dominate at high temperatures.

## 4. Conclusions

This review aimed at presenting a conceptual, first principles analysis of initiation of thermal decomposition reactions in several classes of organic nitro energetic crystals to instigate the development of the molecular theory of initiation of detonation. Despite existing classic macroscale theories [6,136,137,138,139,140] and thousands of papers published in the field of energetic materials, the fundamental understanding of the initiation of chemistry phenomena is limited to micro- and nano-scale. Consequently, significant breakthroughs may be achieved only when chemical and physical mechanisms of materials’ behavior are revealed at the molecular scale.

We reviewed relevant experimental and theoretical literature and augmented the available data by our own simulations to fill the gaps or to test notions recently suggested by researchers. In order to lay groundwork for the future microscale theory, we selected a set of organic energetic materials, nitro ester PETN, nitramine HMX, and C-nitro compounds, TATB, DADNE, and BNFF. Such a set, which combined conventional explosives in use (PETN, HMX, and TATB) with experimental promising energetic materials (DADNE and BNFF), serves to reveal (or confirm and enhance) general trends in chemical decomposition, identify (or supplement and refine) meaningful correlations between structures and properties, and test some novel ideas in the field.

Our modeling of decomposition reactions in the gaseous phase and solid state showed that thermal degradation of explosives is a complex process that often involves several mechanisms triggered with similar activation barriers and progressing with close rates. It is rather unusual that only one reaction would initiate decomposition and continue to progress for a notable time, especially in crystals. One mechanism is typically dominating with another reaction or even two reactions starting somewhat later, which however can switch and become prevailing reactions at higher temperatures. For example, the homolytic loss of NO_2_ will be the dominant decomposition pathway for PETN and HMX at a rather wide range of temperatures with the HONO isomerization proceeding as a slow background reaction. In the case of TATB, the C-NO_2_ bond fission will dominate at high temperature and the CONO isomerization will proceed at higher rates at low temperatures.

The decomposition initiation is comprehensively characterized by a set of descriptors: identified chemical mechanisms, activation barriers, reaction energies, pre-exponential factors, and reactions rates. Only together they reproduce the decomposition picture and if a parameter or two are missing, the lack of data may be misleading. This finding clearly illustrates how hopeless are the attempts to find a single descriptive parameter to measure the sensitivity of materials to initiation and even the thermal decomposition of materials, which represents only one component contributing in sensitivity.

Another factor that strongly affects the decomposition kinetics is a molecular and crystalline structure of the material of interest. We found that the well-accepted correlation between the strength of the critical X-NO_2_ bond and the sensitivity of materials, which roughly follows the series E(C-NO_2_) > E(N-NO_2_) > E(O-NO_2_), is only approximately valid for some isolated molecules (but not all) and certainly it does not work for crystals. Indeed, the difference in the thermal stability of PETN (and most nitro esters) and β-HMX (and most nitramines) can be closely enough explained by a comparison of activation energies required for breaking these critical bonds. The similar approach however cannot be used for analyzing the relative thermal stabilities of HMX polymorphs, β- and δ-phases, or C-nitro compounds, DADNE, TATB and BNFF. The C-NO_2_ bond fission reaction can be largely considered the predominant pathway for decomposition of DADNE and TATB and hence allows for a parallel comparison between these two materials. The stability of BNFF, on the other hand, is mainly determined by the fragmentation mechanisms associated with the cleavage of oxadiazole rings, which require significantly lower activation energy than the C-NO_2_ bond cleavage. Although the ring cleavage reaction is overpowered by the C-NO_2_ loss upon a moderate increase of temperature, the sensitivity of BNFF is higher than what is expected from desired C-nitro materials. This conclusion demonstrates that the chemical composition and the presence of functional groups are as important for defining the stabilization of molecules and materials as the details of the molecular structure and hence may serve as a useful feature for design of new materials with targeted properties and functions.

The crystalline structure and intermolecular interactions impose additional limitations on kinetics and mechanisms of the decomposition of nitro compounds. Mechanisms that may dominate the gas-phase decomposition of single molecules may be restricted or even completely suppressed in the solid state. For example, the recently proposed decomposition mechanism of DADNE via the enamino-imino isomerization followed by the elimination of either CH_2_N_2_ or NO_2_ requires, indeed, a low energy in the gas-phase, which makes this reaction a feasible initiation pathway. However, once the decomposing DADNE molecule is placed on the (010) surface, the overall activation energy of this channel significantly increases due to Coulomb repulsion between neighbor molecules. This precludes DADNE from decomposition through this channel and the C-NO_2_ bond fission becomes the main reaction in the solid-state.

Because energetic materials are densely packed, we predict that there are many other manifestations of the crystal arrangement governing the decomposition chemistry and hence the sensitivity. To mention just a few vivid illustrations, we recall the shear-strain effect in DADNE [21,124,141], the aspects of autocatalysis analyzed for DADNE and TATB [124], polarization-induced charge transfer in δ-HMX [96], enhanced optical absorption on PETN-MgO interfaces [142], and photocatalytic dissociation of PETN molecules adsorbed on F^0^-centers on the α-Al_2_O_3_ (0001) surfaces [143]. A variety of structural and electronic defects also play a role in changing decomposition chemistry in organic molecular crystals [19,122,133,144,145,146]. Without a doubt, these factors have to be carefully analyzed in series of other energetic materials to confirm, refine, and generalize the trends and correlations. Advanced large-scale quantum chemical methods [147,148,149] may need to be involved for such studies, as recently reviewed [150], however they require serious adaptations to become useful for modeling of energetic materials. We would like to emphasize here that partnerships between experimental and theoretical groups, combinations of available methods, and the development of new multi-scale multi-physics techniques are required for studies of the physical and chemical properties of the existing materials and assisting synthesis of novel energetic materials.

Summing up, this review presents a conceptual assessment of thermal decomposition trends in energetic materials that should serve as a solid ground for building the molecular theory of detonation initiation from first principles. By analyzing a large volume of experimental and theoretical studies we would like to emphasize several conclusions and observations. (i) The molecular properties (chemical composition and mutual positions of functional groups in the molecule) define chemistry of individual molecules and therefore provide a useful insight into material’s stability. However by no means conclusions made for gas-phase processes may be extrapolated to materials properties. They are insufficient due to the lack of intermolecular interactions in molecular models; (ii) The realistic quality simulations of materials should take into account the morphology of crystals and defects as they are imperative for reliable predictions and for comparison with experimental studies; (iii) In most energetic materials, the initiation chemistry is defined by the interplay of co-existing reactions that strongly depend upon the chemical composition of the material and its structural arrangement.

## Figures and Tables

**Table 1:** The calculated activation barriers E (kcal/mol), zero-point-energy corrected barriers E_ZPE_ (kcal/mol), and pre-exponential factors, log (*A*, s^−1^) of thermal decomposition of PETN, HMX, DADNE, TATB, and BNFF.

Reaction			Isolated Molecule			(*mnl*) Crystal Surface		
			E	E_ZPE_	log *A*	E	E_ZPE_	log *A*
**PETN** (C_5_H_8_N_4_O_12_)						*(101)*		
*I*	NO_2_^•^ loss		41.8 (41.8) ^a^	36.6 [34.9] b	17.5	40.0 (40.0)	35.3	17.8
*II*	HONO		33.6 (−18.6)	28.8 [40.5]	13.4	34.6 (−10.4)	29.5 [40.9]	14.2
**β-HMX** (C_4_H_8_N_8_O_8_)						*(100)*		
*I*	NO_2_^•^ loss		44.8 (44.8)	40.2 [40.9]	18.3	40.1 (40.1)	37.4	17.3
*II*	HONO		40.4 (−2.4)	35.8 [47.1]	13.4	41.0 (−3.2)	38.1 [47.7]	14.8
**δ-HMX** (C_4_H_8_N_8_O_8_)						*Polar (001)*		
*I*	NO_2_^•^ loss	charge state [0] ^b^	45.1 (45.1)	40.3 [41.5]	18.4	-		
		[+] ^c^	12.7 (−24.3)	- [10.1]	13.4	~20.7 (−6.0)		
		[−] ^c^	17.0 (17.0)	- [15.4]	-	~20.1 (20.1)		
*II*	HONO	[0] ^b^	38.0 (0.7)	33.3 [44.1]	13.3	-		
		[+] ^c^	12.7 (−13.6)	- [10.1]	13.4	~20.7 (12.9)		
**DADNE** (C_2_H_4_N_4_O_4_)						*(010)*		
*I*	NO_2_^•^ loss		69.1 (69.1)	65.4 [68.6]	19.0	66.4 (66.4)	62.3	19.0
*II*	CONO		54.3 (−1.0)	52.1 [61.5]	13.5	55.3 (0.1)	52.6 [64.6]	13.8
*III*	1	CH_2_N_2_ loss (step1)	49.0 (14.7)	45.5 [49.4]	14.3	57.3 (35.2)	53.3 [62.2]	14.0
	2	CH_2_N_2_ loss (step2)	34.0 (14.4)	32.1 [34.4]	14.9	56.5 (38.8)	53.4 [60.7]	15.4
	3	CH_2_N_2_ loss (step3)	44.9 (34.6)	41.0 [47.9]	13.7	52.9 (44.5)	48.6 [58.8]	15.2
	4	NO_2_^•^ loss from AIDNE	50.7 (50.7)	46.2 [47.8]	19.1	67.1 (67.1)	61.9	19.3
**TATB** (C_6_H_6_N_6_O_6_)						*(001)*		
*I*	NO_2_^•^ loss		74.9 (74.9)	70.7 [65.8]	18	75.0 (75.0)	71.1	19
*II*	CONO		54.9 (4.3)	52.5 [58.6]	13.1	54.0 (5.6)	51.5 [58.0]	13.8
*III*	1	H_2_O loss (step1)	44.5 (37.5)	43.2 [42.1]	13.0	49.0 (43.0)	47.3 [46.1]	13.8
	2	H_2_O loss (step2)	65.3 (40.9)	62.6 [68.6]	13.4	74.6 (44.5)	71.3 [74.3]	14.7
	3	H_2_O loss (step3)	52.8 (41.7)	47.5 [58.8]	13.3	59.1 (49.5)	53.2 [65.5]	14.3
	4	HONO loss	95.4 (42.5)	91.3 [94.1]	13.4	-	-	-
**BNFF** (C_6_N_8_O_8_)						*(001)*		
*I*	NO_2_^•^ loss		63.2 (63.2)	59.3 [56.2]	17.9	60.1 (60.1)	56.2	18.4
*II*	CONO		49.5 (−9.5)	47.2 [54.3]	13.4	49.1 (−11.0)	46.8 [51.4]	13.3
*III*	RC CN_2_O_3_		48.7 (36.4)	45.7 [50.5]	15.2	48.0 (31.7)	45.1 [49.4]	15.5
*IV*	RC CN_2_O_2_		47.1 (36.6)	44.0 [48.9]	16.3	44.2 (31.2)	41.4 [45.8]	15.4

^a^ The corresponding reaction energies are shown in parentheses; ^b^ Activation barriers and reaction energies obtained with hybrid PBE0 (for PETN, HMX, and DADNE) and B3LYP (for BNFF) functionals are shown in square brackets; ^c^ Activation barriers and reaction energies for decomposition reactions of ion radicals were obtained using M06 functional.

## Supplementary Materials

Details of periodic calculations of DADNE and TATB crystals and their surfaces are included. Supplementary materials can be accessed at: http://www.mdpi.com/1420-3049/21/2/236/s1.
