# Supplementary material for: Molecular Theory of Detonation Initiation: Insight from First Principles Modeling of the Decomposition Mechanisms of Organic Nitro Energetic Materials
Source: Molecules. 2016 Feb 19;21(2):236. doi: 10.3390/molecules21020236 (PMC6273078; doi:10.3390/molecules21020236)
Supplement: Supplementary file 1 [file molecules-21-00236-s001.pdf]

Supplementary Materials: Molecular Theory of Detonation Initiation: Insight from First Principles Modeling of the Decomposition Mechanisms of Organic Nitro Energetic Materials

Roman V. Tsyshevsky, Onise Sharia and Maija M. Kuklja

1. Modeling DADNE

In simulating an ideal DADNE crystal, we used 2 × 2 × 2 Monkhorst-Pack k-point mesh and the kinetic energy cut-off was set to 520 eV. Atomic coordinates and lattice constants were allowed to simultaneously relax without any symmetry constraints. The convergence criterion for electronic steps was set to 10^{-5} eV, and the maximum force acting on any atom was set not to exceed 0.02 eV/Å. The calculated lattice constants, a = 7.11 Å, b = 6.83 Å c = 11.558 Å, were found to be in agreement with the experimental lattice constants a = 6.941 Å, b = 6.569 Å and c = 11.56 Å [116] within 4%.

Surface reactions were simulated in a slab model, in which the supercell with lattice consisted of the four-molecule-thick surface layer (appropriately cut out to create the (010) surface) on top of a 10 Å of vacuum. Surface supercell calculations we performed at the Gamma point only, with kinetic energy cut-off set to 520 eV. The convergence criteria for electronic and ionic steps were set to 10^{-5} eV and 0.03 Å/eV, respectively. Supercell has lattice parameters a = 14.220 Å, b = 22.429 Å and c = 23.119 Å and contains 32 molecules. Coordinates of unit cell and surface supercell are collected below.

2. Modeling TATB

In simulating an ideal TATB crystal, we used 2 × 2 × 3 Monkhorst-Pack k-point mesh and the kinetic energy cut-off was set to 520 eV. Atomic coordinates and lattice constants were allowed to simultaneously relax without any symmetry constraints. The convergence criterion for electronic steps was set to 10^{-5} eV, and the maximum force acting on any atom was set not to exceed 0.02 eV/Å. The calculated lattice constants, a = 9.207 Å, b = 9.232 Å c = 7.163 Å, were found to be in agreement with the experimental lattice constants a = 9.010 Å, b = 9.028 Å and c = 6.812 Å [1] within 5%.

Surface reactions were simulated in a slab model, in which the supercell with lattice consisted of the four-molecule-thick surface layer (appropriately cut out to create the (001) surface) on top of a 10 Å of vacuum. Surface supercell calculations we performed at the Gamma point only, with kinetic energy cut-off set to 520 eV. The convergence criteria for electronic and ionic steps were set to 10^{-5} eV and 0.03 Å/eV, respectively. Supercell has lattice parameters a = 18.433 Å, b = 15.971 Å and c = 20.176 Å and contains 16 molecules. Coordinates of unit cell and surface supercell are collected below.

3. Coordinates

3.1. DADNE unit cell

1.0

7.1101876328689233	−0.0005603435561343	−0.0486170540142459
-0.0005365011028712	6.8334751457089720	0.0000845972268726
-0.2032549274931936	0.0001876858325154	11.5579975699804454

H C N O
16 8 16 16

Direct

0.7463286176948524	0.3322607512022090	0.3578620824202476
0.9452780760099621	0.2946062035375151	0.4500150617998909
0.4530670804250810	0.2940101277832702	0.4248557655810541

0.4174665741384880	0.2020352600710774	0.5650886174963731
0.7536751555801490	0.8322724908668696	0.1421381493282493
0.5547239859062347	0.7946278975967039	0.0499831543573485
0.0469225107147145	0.7940074575140387	0.0751487501000364
0.0824965217975275	0.7020290936136936	0.9349232076325854
0.2536713823051479	0.6677392487977907	0.6421379175797526
0.0547219239900380	0.7053937964624847	0.5499849382001094
0.5469329195749194	0.7059898722167303	0.5751442344189456
0.5825334258615122	0.7979647399289225	0.4349113825036275
0.2463248444198511	0.1677275091331306	0.8578618506717508
0.4452760140937654	0.2053721024032957	0.9500168456426521
0.9530774892852854	0.2059925424859541	0.9248512498999564
0.9175034782024726	0.2979709063863067	0.0650767923674217
0.6917560763008982	0.2443409428759436	0.5225325000404096
0.7704256623607451	0.1888816289454133	0.6361348957926580
0.8082315415710665	0.7443412315979270	0.9774625156764805
0.7295400322744265	0.6888913690416713	0.8638801656772148
0.3082439236991017	0.7556590571240565	0.4774674999595978
0.2295743376392549	0.8111183710545865	0.3638651042073419
0.1917684584289332	0.2556587684020733	0.0225374843235191
0.2704599677255735	0.3111086309583283	0.1361198343227852
0.8031563185551199	0.2930673956200472	0.4365420296709465
0.5060514642234891	0.2536694322951756	0.5042833030097399
0.9650932742513625	0.1676970137231310	0.6586590401303306
0.6491807397496517	0.1564527246410012	0.7300191060749780
0.6968186873244014	0.7930819347678737	0.0634658605891840
0.9939356992270466	0.7536715880303883	0.9957272759620465
0.5348568332039043	0.6676940275822685	0.8413440571517474
0.8507969912304956	0.6564562408540047	0.7699805484538806
0.1968436814448800	0.7069326043799525	0.5634579703290534
0.4939485357765109	0.7463305677048243	0.4957166969902604
0.0349067257486377	0.8323029862768695	0.3413409598696690
0.3508192602503487	0.8435472753589991	0.2699808939250217
0.3031813126755989	0.2069180652321257	0.9365341394108159
0.0060643007729534	0.2463284119696118	0.0042727240379538
0.4651431667960961	0.3323059724177312	0.1586559428482600
0.1492030087695037	0.3435437591459952	0.2300194515461194
0.0233187209848485	0.0905683727525736	0.7515802142606762
0.0784360570544222	0.2186632859642228	0.5819055228271341
0.7028824929116996	0.1929790542203638	0.8317862067187910
0.4851680139801969	0.0951120514277908	0.7084389115704258
0.4766251473171835	0.5905596279324418	0.7484361152137051
0.4215499716445278	0.7186649460478047	0.9180935483840033
0.7970756790144982	0.6929802252672498	0.6682155069357761
0.0147654072193283	0.5951435753380045	0.7915643079607619
0.9766812790151517	0.9094316272474264	0.2484197857393239
0.9215639429455778	0.7813367140357771	0.4180944771728661
0.2971175070882998	0.8070209457796360	0.1682137932812095
0.5148319860198031	0.9048879485722163	0.2915610884295747
0.5233748526828160	0.4094403720675583	0.2515638847862947
0.5784500283554720	0.2813350539521955	0.0819064516159966
0.2029243209855017	0.3070197747327502	0.3317844930642233

0.9852345927806719 0.4048564246619959 0.2084356920392379

3.2. DADNE (010) surface

1.0

14.2207002639999995 0.0000000000000000 0.0000000000000000
0.0000000000000000 22.4291992188000009 0.0000000000000000
-0.5645396563000000 0.0000000000000000 23.1127067394999983
H C N O
128 64 128 128

Direct

0.3731145836938200 0.0565531168162792 0.1775109354949851
0.3731145836747846 0.0565531168162792 0.6775109205789523
0.8731145836938200 0.0565531168162792 0.1775109354949851
0.8731145836747846 0.0565531168162792 0.6775109205789523
0.3737942507643455 0.3807182857606092 0.1776382272492062
0.3737942507615886 0.3807182857606092 0.6776382570271996
0.8737942507643455 0.3807182857606092 0.1776382272492062
0.8737942507615886 0.3807182857606092 0.6776382570271996
0.4725684207883406 0.0474558204403232 0.2241764797369936
0.4725684207975990 0.0474558204403232 0.7241764797045431
0.9725683909726529 0.0474558204403232 0.2241764797369936
0.9725683909819113 0.0474558204403232 0.7241764797045431
0.4732128480062682 0.3700319549163523 0.2238397692162616
0.4732128479889523 0.3700319549163523 0.7238397543434938
0.9732128778219559 0.3700319549163523 0.2238397692162616
0.9732128778046399 0.3700319549163523 0.7238397543434938
0.2260552029891708 0.0426300738440759 0.2106906309319896
0.2260552030001416 0.0426300738440759 0.7106906309428040
0.7260552029891708 0.0426300738440759 0.2106906309319896
0.7260552030001416 0.0426300738440759 0.7106906309428040
0.2271588859944630 0.3703849686130615 0.2112075963021679
0.2271588859934327 0.3703849686130615 0.7112076261234263
0.7271588412709279 0.3703849686130615 0.2112075963021679
0.7271588412698976 0.3703849686130615 0.7112076261234263
0.2092320260670999 0.0148711564745850 0.2809913841609602
0.2092320260197706 0.0148711564745850 0.7809913543613379
0.7092320111592525 0.0148711564745850 0.2809913841609602
0.7092320111119232 0.0148711564745850 0.7809913543613379
0.2088409232084336 0.3436890205299182 0.2816690674454674
0.2088409232297002 0.3436890205299182 0.7816690376025801
0.7088409083005863 0.3436890205299182 0.2816690674454674
0.7088409083218600 0.3436890205299182 0.7816690376025801
0.3765228674209800 0.2193021039292447 0.0724932184479883
0.3765228673736508 0.2193021039292447 0.5724931886483589
0.8765228376052925 0.2193021039292447 0.0724932184479883
0.8765228375579632 0.2193021039292447 0.5724931886483589
0.3758742194204110 0.5454850943808641 0.0725968567485830
0.3758742193893674 0.5454850943808641 0.5725968716429869
0.8758741896047235 0.5454850943808641 0.0725968567485830
0.8758741896439952 0.5454850943808641 0.5725968716429869

0.2771891805699406	0.2088041063293285	0.0260959440690349
0.2771891805544436	0.2088041063293285	0.5260959310134445
0.7771891805699406	0.2088041063293285	0.0260959440690349
0.7771891805544365	0.2088041063293285	0.5260959310134445
0.2763934931728751	0.5350009746311624	0.0263764690125431
0.2763934931453629	0.5350009746311624	0.5263764857673966
0.7763934931728752	0.5350009746311624	0.0263764690125431
0.7763934931453630	0.5350009746311624	0.5263764857673966
0.5233272780472635	0.2083983174787505	0.0390657253723159
0.5233272780494449	0.2083983174787505	0.5390657216189733
0.0233273414056061	0.2083983174787505	0.0390657253723159
0.0233273414077875	0.2083983174787505	0.5390657216189733
0.5226664168891543	0.5361356010421517	0.0389530342869376
0.5226664168718383	0.5361356010421517	0.5389530194141626
0.0226664485331679	0.5361356010421517	0.0389530342869376
0.0226664485158449	0.5361356010421517	0.5389530194141626
0.5414568980007659	0.1820859565682446	0.4683791176515758
0.5414568980117367	0.1820859565682446	0.9683791176623904
0.0414569054546862	0.1820859565682446	0.4683791176515758
0.0414569054656640	0.1820859565682446	0.9683791176623904
0.5409774809184765	0.5087983251157784	0.4687244619654911
0.5409774809157196	0.5087983251157784	0.9687244917434844
0.0409774697375892	0.5087983251157784	0.4687244619654911
0.0409774697348394	0.5087983251157784	0.9687244917434844
0.1259342620165229	0.1739434180538823	0.3226736132710166
0.1259342620154855	0.1739434180538823	0.8226736430922750
0.6259342471086756	0.1739434180538823	0.3226736132710166
0.6259342471076453	0.1739434180538823	0.8226736430922750
0.1268634481722833	0.4973188845345549	0.3221192814228712
0.1268634481935570	0.4973188845345549	0.8221192515799769
0.6268634928958184	0.4973188845345549	0.3221192814228712
0.6268634928467697	0.4973188845345549	0.8221192515799769
0.0268017520100389	0.1849856945337695	0.2763009665708629
0.0268017520330250	0.1849856945337695	0.7763009367712405
0.5268017464547530	0.1849856945337695	0.2763009665708629
0.5268017464074237	0.1849856945337695	0.7763009367712405
0.0276128329120988	0.5068433878271692	0.2753002585971668
0.0276128329350920	0.5068433878271692	0.7753002287975375
0.5276128440929861	0.5068433878271692	0.2753002585971668
0.5276128441159722	0.5068433878271692	0.7753002287975375
0.2729075797349722	0.1835477838266662	0.2891205428767689
0.2729075796739153	0.1835477838266662	0.7891205428443183
0.7729075797349723	0.1835477838266662	0.2891205428767689
0.7729075796739153	0.1835477838266662	0.7891205428443183
0.2742642389968834	0.5111420345329062	0.2890608228661291
0.2742642390198766	0.5111420345329062	0.7890607930665067
0.7742642389968835	0.5111420345329062	0.2890608228661291
0.7742642390198766	0.5111420345329062	0.7890607930665067
0.2914355698705902	0.2096755131686552	0.2184909143260144
0.2914355698815681	0.2096755131686552	0.7184909143368359
0.7914355698705903	0.2096755131686552	0.2184909143260144
0.7914355698815682	0.2096755131686552	0.7184909143368359

0.2911878330584288	0.5393349579535013	0.2189416724006933
0.2911878330676871	0.5393349579535013	0.7189416723682428
0.7911878032427340	0.5393349579535013	0.2189416724006933
0.7911878032519923	0.5393349579535013	0.7189416723682428
0.1247999332736069	0.0088202993495184	0.4272676331142267
0.1247999332845848	0.0088202993495184	0.9272676331250482
0.6247999332736069	0.0088202993495184	0.4272676331142267
0.6247999332845848	0.0088202993495184	0.9272676331250482
0.1229068987322532	0.3351965699049465	0.4274786677645232
0.1229068986849239	0.3351965699049465	0.9274786379649008
0.6229068614626454	0.3351965699049465	0.4274786677645232
0.6229068614856315	0.3351965699049465	0.9274786379649008
0.2239304486009770	0.0191665025770489	0.4738088747170429
0.2239304486239702	0.0191665025770489	0.9738088449174205
0.7239304038774420	0.0191665025770489	0.4738088747170429
0.7239304039004352	0.0191665025770489	0.9738088449174205
0.2223011079122085	0.3456346665126563	0.4738820597672725
0.2223011079094516	0.3456346665126563	0.9738820895452587
0.7223011079122086	0.3456346665126563	0.4738820597672725
0.7223011079094517	0.3456346665126563	0.9738820895452587
0.9777289865356823	0.0178926981149203	0.4608110426177308
0.9777289865346449	0.0178926981149203	0.9608110724389891
0.4777289865356822	0.0178926981149203	0.4608110426177308
0.4777289865346449	0.0178926981149203	0.9608110724389891
0.9760857141318211	0.3458171370201026	0.4610847692791091
0.9760857141427990	0.3458171370201026	0.9610847692899236
0.4760857141318211	0.3458171370201026	0.4610847692791091
0.4760857141427990	0.3458171370201026	0.9610847692899236
0.9590391367729841	0.0451736379702326	0.0309917733868750
0.9590391368065857	0.0451736379702326	0.5309917491686014
0.4590391069572893	0.0451736379702326	0.0309917733868750
0.4590391069908909	0.0451736379702326	0.5309917491686014
0.9579986700160504	0.3717683785355557	0.0318382373917900
0.9579986699850068	0.3717683785355557	0.5318382522861940
0.4579986700160506	0.3717683785355557	0.0318382373917900
0.4579986699850070	0.3717683785355557	0.5318382522861940
0.3459637545900496	0.0299545312290735	0.2599932452461822
0.3459637546010275	0.0299545312290735	0.7599932452569966
0.8459637844057445	0.0299545312290735	0.2599932452461822
0.8459637844167224	0.0299545312290735	0.7599932452569966
0.3461783066809924	0.3559164473720426	0.2604583447807925
0.3461783066799550	0.3559164473720426	0.7604583746020509
0.8461782768652976	0.3559164473720426	0.2604583447807925
0.8461782768642673	0.3559164473720426	0.7604583746020509
0.3852932483525189	0.0154369312461557	0.3172864906555122
0.3852932483051896	0.0154369312461557	0.8172864608558899
0.8852932780978843	0.0154369312461557	0.3172864906555122
0.8852932781208774	0.0154369312461557	0.8172864608558899
0.3852967665409908	0.3401958555561894	0.3175829179621146
0.3852967664816463	0.3401958555561894	0.8175829179729289
0.8852967367252961	0.3401958555561894	0.3175829179621146
0.8852967367362740	0.3401958555561894	0.8175829179729289

0.4042616277820124	0.1943006699723774	0.4897150764947805
0.4042616277912707	0.1943006699723774	0.9897150764623300
0.9042615681506299	0.1943006699723774	0.4897150764947805
0.9042615681598882	0.1943006699723774	0.9897150764623300
0.4036970055652604	0.5209526710793880	0.4897085560188106
0.4036970055745188	0.5209526710793880	0.9897085559863601
0.9036969757495655	0.5209526710793880	0.4897085560188106
0.9036969757588310	0.5209526710793880	0.9897085559863601
0.3651183331543669	0.1789367481528719	0.4324571499338719
0.3651183331773601	0.1789367481528719	0.9324571201342495
0.8651183331543669	0.1789367481528719	0.4324571499338719
0.8651183331773601	0.1789367481528719	0.9324571201342495
0.3649364125761318	0.5045252808076475	0.4325833313276637
0.3649364125733820	0.5045252808076475	0.9325833611056499
0.8649364423918194	0.5045252808076475	0.4325833313276637
0.8649364423890696	0.5045252808076475	0.9325833611056499
0.1540184475558989	0.1982756937738809	0.2397780077487497
0.1540184475531491	0.1982756937738809	0.7397780375267431
0.6540184475558990	0.1982756937738809	0.2397780077487497
0.6540184475531492	0.1982756937738809	0.7397780375267431
0.1544379392907989	0.5242698437573944	0.2397851638116688
0.1544379392314543	0.5242698437573944	0.7397851638224833
0.6544379392907987	0.5242698437573944	0.2397851638116688
0.6544379392314542	0.5242698437573944	0.7397851638224833
0.1151023263260348	0.2138490588206871	0.1825750115752822
0.1151023263370127	0.2138490588206871	0.6825750115861038
0.6151023486878024	0.2138490588206871	0.1825750115752822
0.6151023486987803	0.2138490588206871	0.6825750115861038
0.1151808836011988	0.5391178632677182	0.1825423385337130
0.1151808835838828	0.5391178632677182	0.6825423236609451
0.6151808836011988	0.5391178632677182	0.1825423385337130
0.6151808835838829	0.5391178632677182	0.6825423236609451
0.0965322184027917	0.0329530146496448	0.0101873386190855
0.0965322183590009	0.0329530146496448	0.5101873106799126
0.5965322333106390	0.0329530146496448	0.0101873386190855
0.5965322332668482	0.0329530146496448	0.5101873106799126
0.0953133368531023	0.3598894240824914	0.0103738653308139
0.0953133368895389	0.3598894240824914	0.5103738606688791
0.5953133219452549	0.3598894240824914	0.0103738653308139
0.5953133219816915	0.3598894240824914	0.5103738606688791
0.1353506028585316	0.0493868881594390	0.0672787257369939
0.1353506028536431	0.0493868881594390	0.5672787182627521
0.6353506177663719	0.0493868881594390	0.0672787257369939
0.6353506177614905	0.0493868881594390	0.5672787182627521
0.1345233035631974	0.3750678394828624	0.0676335356397494
0.1345233035883222	0.3750678394828624	0.5676335430923550
0.6345232886553500	0.3750678394828624	0.0676335356397494
0.6345232886804748	0.3750678394828624	0.5676335430923550
0.4016987388374171	0.0448854661101868	0.2168978180185477
0.4016987387900878	0.0448854661101868	0.7168977882189254
0.9016987090217294	0.0448854661101868	0.2168978180185477
0.9016987090447155	0.0448854661101868	0.7168977882189254

0.4021269867966237	0.3693901813731844	0.2171714508060849
0.4021269867938668	0.3693901813731844	0.7171714805840783
0.9021269272355565	0.3693901813731844	0.2171714508060849
0.9021269272328067	0.3693901813731844	0.7171714805840783
0.2531599287474213	0.0300816212147466	0.2501586761439629
0.2531599287583992	0.0300816212147466	0.7501586761547773
0.7531599585631160	0.0300816212147466	0.2501586761439629
0.7531599585740939	0.0300816212147466	0.7501586761547773
0.2533485437169682	0.3589955945150718	0.2512325516095550
0.2533485437159380	0.3589955945150718	0.7512325814308134
0.7533485437169682	0.3589955945150718	0.2512325516095550
0.7533485437159380	0.3589955945150718	0.7512325814308134
0.4827908056425146	0.0111468916289846	0.3291907357054635
0.4827908056655077	0.0111468916289846	0.8291907059058339
0.9827908652738970	0.0111468916289846	0.3291907357054635
0.9827908652968902	0.0111468916289846	0.8291907059058339
0.4825288849697426	0.3342540645491340	0.3292223306474006
0.4825288849807205	0.3342540645491340	0.8292223306582222
0.9825289147854375	0.3342540645491340	0.3292223306474006
0.9825289147964154	0.3342540645491340	0.8292223306582222
0.3245947867653991	0.0065306253616755	0.3645496635766152
0.3245947867060546	0.0065306253616755	0.8645496635874297
0.8245947867653990	0.0065306253616755	0.3645496635766152
0.8245947867060545	0.0065306253616755	0.8645496635874297
0.3244455125258447	0.3309787770695737	0.3646128998676387
0.3244455124647878	0.3309787770695737	0.8646128998351883
0.8244455422712170	0.3309787770695737	0.3646128998676387
0.8244455422804754	0.3309787770695737	0.8646128998351883
0.3482408306018114	0.2079111191861925	0.0329569771873236
0.3482408306318744	0.2079111191861925	0.5329569511086003
0.8482408007861166	0.2079111191861925	0.0329569771873236
0.8482408008161797	0.2079111191861925	0.5329569511086003
0.3475382516244048	0.5345766183164548	0.0328918499713437
0.3475382516265862	0.5345766183164548	0.5328918462179940
0.8475382218087099	0.5345766183164548	0.0328918499713437
0.8475382218108983	0.5345766183164548	0.5328918462179940
0.4970619196027679	0.1968418843113675	0.4991207949037462
0.4970619196257611	0.1968418843113675	0.9991207651041167
0.9970618897870801	0.1968418843113675	0.4991207949037462
0.9970618898100733	0.1968418843113675	0.9991207651041167
0.4964823006877843	0.5245435161381797	0.4989529216502989
0.4964823006987551	0.5245435161381797	0.9989529216611204
0.9964823305034719	0.5245435161381797	0.4989529216502989
0.9964823305144498	0.5245435161381797	0.9989529216611204
0.2678016498075558	0.1727182503578149	0.4209196488912283
0.2678016498305490	0.1727182503578149	0.9209196190916059
0.7678016498075558	0.1727182503578149	0.4209196488912283
0.7678016498305490	0.1727182503578149	0.9209196190916059
0.2675639307830996	0.4995573807983957	0.4206550080983508
0.2675639307803498	0.4995573807983957	0.9206550378763443
0.7675638712220395	0.4995573807983957	0.4206550080983508
0.7675638712192897	0.4995573807983957	0.9206550378763443

0.4258631586092739	0.1705036177175583	0.3853032607605224
0.4258631585619446	0.1705036177175583	0.8853032309609000
0.9258631586092738	0.1705036177175583	0.3853032607605224
0.9258631585619446	0.1705036177175583	0.8853032309609000
0.4259978041773096	0.4938619022655393	0.3859341840324672
0.4259978041882875	0.4938619022655393	0.8859341840432816
0.9259978041773096	0.4938619022655393	0.3859341840324672
0.9259978041882875	0.4938619022655393	0.8859341840432816
0.0978583758421729	0.1854823747101627	0.2831354531112660
0.0978583759097455	0.1854823747101627	0.7831354828892524
0.5978583684585752	0.1854823747101627	0.2831354531112660
0.5978583684558183	0.1854823747101627	0.7831354828892524
0.0984974082132033	0.5091929021350300	0.2827249781777535
0.0984974082361893	0.5091929021350300	0.7827249483781312
0.5984973933053558	0.5091929021350300	0.2827249781777535
0.5984973933283489	0.5091929021350300	0.7827249483781312
0.2468083723832658	0.1946239026901551	0.2489831058213873
0.2468083723522222	0.1946239026901551	0.7489831207157912
0.7468083574754255	0.1946239026901551	0.2489831058213873
0.7468083574443748	0.1946239026901551	0.7489831207157912
0.2472429397653456	0.5238890542840553	0.2496448787299334
0.2472429397180093	0.5238890542840553	0.7496448489303040
0.7472429844185584	0.5238890542840553	0.2496448787299334
0.7472429844415516	0.5238890542840553	0.7496448489303040
0.0179027157594627	0.2199056843828465	0.1708036621781921
0.0179027157584324	0.2199056843828465	0.6708036919994504
0.5179026971246553	0.2199056843828465	0.1708036621781921
0.5179026971236250	0.2199056843828465	0.6708036919994504
0.0177129500199473	0.5436137744740684	0.1705845775926364
0.0177129499888966	0.5436137744740684	0.6705845924870403
0.5177129593021933	0.5436137744740684	0.1705845775926364
0.5177129593414650	0.5436137744740684	0.6705845924870403
0.1761680842523294	0.2227218741902936	0.1355905920549406
0.1761680842933206	0.2227218741902936	0.6355906069926095
0.6761680395991096	0.2227218741902936	0.1355905920549406
0.6761680395697784	0.2227218741902936	0.6355906069926095
0.1760309542686286	0.5479975285646235	0.1353450320439518
0.1760309542916218	0.5479975285646235	0.6353450022443223
0.6760309542686287	0.5479975285646235	0.1353450320439518
0.6760309542916219	0.5479975285646235	0.6353450022443223
0.1528446010222622	0.0193310643123941	0.4671061556186069
0.1528446010332401	0.0193310643123941	0.9671061556294285
0.6528445861144220	0.0193310643123941	0.4671061556186069
0.6528445861253928	0.0193310643123941	0.9671061556294285
0.1512724173528555	0.3466126480701865	0.4669937194196273
0.1512724173038067	0.3466126480701865	0.9669936895767329
0.6512724321903806	0.3466126480701865	0.4669937194196273
0.6512724322116542	0.3466126480701865	0.9669936895767329
0.0037525409787774	0.0294321702862975	0.0008653059716379
0.0037525409618665	0.0294321702862975	0.5008652981945344
0.5037525316262089	0.0294321702862975	0.0008653059716379
0.5037525316092980	0.0294321702862975	0.5008652981945344

0.0024888211639530	0.3571098520502454	0.0010899512180277
0.0024888211832230	0.3571098520502454	0.5010899762367277
0.5024888593477309	0.3571098520502454	0.0010899512180277
0.5024888593670008	0.3571098520502454	0.5010899762367277
0.2326307618475457	0.0548577574603695	0.0791525535087028
0.2326307618585235	0.0548577574603695	0.5791525535195243
0.7326307469396983	0.0548577574603695	0.0791525535087028
0.7326307469506762	0.0548577574603695	0.5791525535195243
0.2318042910509490	0.3809930961413594	0.0792130668770595
0.2318042911039555	0.3809930961413594	0.5792130520042914
0.7318042762134240	0.3809930961413594	0.0792130668770595
0.7318042761961081	0.3809930961413594	0.5792130520042914
0.0743656180709731	0.0597765218945341	0.1140685718725851
0.0743656180399294	0.0597765218945341	0.6140685867669889
0.5743655733474380	0.0597765218945341	0.1140685718725851
0.5743655733163944	0.0597765218945341	0.6140685867669889
0.0737731175314286	0.3838526317049520	0.1147678435012144
0.0737731175724198	0.3838526317049520	0.6147678584388833
0.5737731175314286	0.3838526317049520	0.1147678435012144
0.5737731175724198	0.3838526317049520	0.6147678584388833
0.5119545645059904	0.9884907515184297	0.3760185054536514
0.5119545645169683	0.9884907515184297	0.8760185054644659
0.0119546231528947	0.9884907515184297	0.3760185054536514
0.0119546231638725	0.9884907515184297	0.8760185054644659
0.5113740947132586	0.3114679571037562	0.3761034998438131
0.5113740947242364	0.3114679571037562	0.8761034998546277
0.0113740137045868	0.3114679571037562	0.3761034998438131
0.0113740136452423	0.3114679571037562	0.8761034998546277
0.5394025030782549	0.0276513365502064	0.2912079199826049
0.5394025030772175	0.0276513365502064	0.7912079498038633
0.0394025477314675	0.0276513365502064	0.2912079199826049
0.0394025477304373	0.0276513365502064	0.7912079498038633
0.5394205071733874	0.3493859844018137	0.2908738679210041
0.5394205071843653	0.3493859844018137	0.7908738679318185
0.0394205817126173	0.3493859844018137	0.2908738679210041
0.0394205817235951	0.3493859844018137	0.7908738679318185
0.3504748285234375	0.0209522167211515	0.4147451695251952
0.3504748285326958	0.0209522167211515	0.9147451694927446
0.8504748285234375	0.0209522167211515	0.4147451695251952
0.8504748285326958	0.0209522167211515	0.9147451694927446
0.3506363908114621	0.3437320143402772	0.4152221827890312
0.3506363908087052	0.3437320143402772	0.9152222125670175
0.8506363311800795	0.3437320143402772	0.4152221827890312
0.8506363312476450	0.3437320143402772	0.9152222125670175
0.2437501293970399	0.9859128751518054	0.3541853699071455
0.2437501293960025	0.9859128751518054	0.8541853997284037
0.7437501443048803	0.9859128751518054	0.3541853699071455
0.7437501443038500	0.9859128751518054	0.8541853997284037
0.2430202462624420	0.3112864215363813	0.3539396666403632
0.2430202462717004	0.3112864215363813	0.8539396666079128
0.7430202165170697	0.3112864215363813	0.3539396666403632
0.7430202165263281	0.3112864215363813	0.8539396666079128

0.2389291664032889	0.1499848172656810	0.3740414815257064
0.2389291664142668	0.1499848172656810	0.8740414815365207
0.7389291962189766	0.1499848172656810	0.3740414815257064
0.7389291962299545	0.1499848172656810	0.8740414815365207
0.2385686881942954	0.4757711681563531	0.3742827791196671
0.2385686882052733	0.4757711681563531	0.8742827791304817
0.7385686732864550	0.4757711681563531	0.3742827791196671
0.7385686732974329	0.4757711681563531	0.8742827791304817
0.2109302039773810	0.1876313165097585	0.4593917779795187
0.2109302039180364	0.1876313165097585	0.9593917779903330
0.7109302337227463	0.1876313165097585	0.4593917779795187
0.7109302337337242	0.1876313165097585	0.9593917779903330
0.2106399429850160	0.5169644522101464	0.4581088884042944
0.2106399429942744	0.5169644522101464	0.9581088883718440
0.7106399131693284	0.5169644522101464	0.4581088884042944
0.7106399131785868	0.5169644522101464	0.9581088883718440
0.3990690185933295	0.1832210077622886	0.3348081972552193
0.3990690186043074	0.1832210077622886	0.8348081972660408
0.8990690185933293	0.1832210077622886	0.3348081972552193
0.8990690186043072	0.1832210077622886	0.8348081972660408
0.3997956839996736	0.5045706264212150	0.3349301047915980
0.3997956840226596	0.5045706264212150	0.8349300749919757
0.8997956839996736	0.5045706264212150	0.3349301047915980
0.8997956840226596	0.5045706264212150	0.8349300749919757
0.5078730324254480	0.1516767337077613	0.3957166648315462
0.5078730323661035	0.1516767337077613	0.8957166648423607
0.0078731199738840	0.1516767337077613	0.3957166648315462
0.0078731199145395	0.1516767337077613	0.8957166648423607
0.5078123901474134	0.4750297807148284	0.3973527281496724
0.5078123901583913	0.4750297807148284	0.8973527281604938
0.0078124255887024	0.4750297807148284	0.3973527281496724
0.0078124255996802	0.4750297807148284	0.8973527281604938
0.9892847743535612	0.2424390800351256	0.1237607019837698
0.9892847743786860	0.2424390800351256	0.6237607094363826
0.4892847743535612	0.2424390800351256	0.1237607019837698
0.4892847743786859	0.2424390800351256	0.6237607094363826
0.9887723715847410	0.5660722931403133	0.1236303387753852
0.9887723715515586	0.5660722931403133	0.6236303164175540
0.4887723715847410	0.5660722931403133	0.1236303387753852
0.4887723715515586	0.5660722931403133	0.6236303164175540
0.9608493645570766	0.2050488814951199	0.2091691611854948
0.9608493644977392	0.2050488814951199	0.7091691611963092
0.4608493347413889	0.2050488814951199	0.2091691611854948
0.4608493347523668	0.2050488814951199	0.7091691611963092
0.9609177115966085	0.5274611028110295	0.2086081893482148
0.9609177115775731	0.5274611028110295	0.7086081744321748
0.4609177115966087	0.5274611028110295	0.2086081893482148
0.4609177115775733	0.5274611028110295	0.7086081744321748
0.1499427673447175	0.2100845530241937	0.0849692840989154
0.1499427673677036	0.2100845530241937	0.5849692542992930
0.6499427673447176	0.2100845530241937	0.0849692840989154
0.6499427673677036	0.2100845530241937	0.5849692542992930

0.1502644286826493	0.5337613452705307	0.0850750318373350
0.1502644287197894	0.5337613452705307	0.5850750094795040
0.6502644584983370	0.5337613452705307	0.0850750318373350
0.6502644585354771	0.5337613452705307	0.5850750094795040
0.2578230565565633	0.2420184361834155	0.1463227541611397
0.2578230565795565	0.2420184361834155	0.6463227243615101
0.7578230863722583	0.2420184361834155	0.1463227541611397
0.7578230863952443	0.2420184361834155	0.6463227243615101
0.2569586766640051	0.5684291616599546	0.1458815421323595
0.2569586766749830	0.5684291616599546	0.6458815421431741
0.7569586468483103	0.5684291616599546	0.1458815421323595
0.7569586468592882	0.5684291616599546	0.6458815421431741
0.2613900699099783	0.0787664437747962	0.1255326153224118
0.2613900698909429	0.0787664437747962	0.6255326004063789
0.7613900699099784	0.0787664437747962	0.1255326153224118
0.7613900698909429	0.0787664437747962	0.6255326004063789
0.2607539825112591	0.4035279929656679	0.1261506229185653
0.2607539824922236	0.4035279929656679	0.6261506080025253
0.7607540420723191	0.4035279929656679	0.1261506229185653
0.7607540421236061	0.4035279929656679	0.6261506080025253
0.2897286845739659	0.0377618240345985	0.0416437794988793
0.2897286845407836	0.0377618240345985	0.5416437571410481
0.7897286845739661	0.0377618240345985	0.0416437794988793
0.7897286845407837	0.0377618240345985	0.5416437571410481
0.2886945951845780	0.3660415260808352	0.0407773860810991
0.2886945951372487	0.3660415260808352	0.5407773562814696
0.7886945951845780	0.3660415260808352	0.0407773860810991
0.7886945951372487	0.3660415260808352	0.5407773562814696
0.1005790145680847	0.0486779399672289	0.1650015641885544
0.1005790145387535	0.0486779399672289	0.6650015791262233
0.6005789847523970	0.0486779399672289	0.1650015641885544
0.6005789847230658	0.0486779399672289	0.6650015791262233
0.1003486866199354	0.3709813049583744	0.1652653902440322
0.1003486866009000	0.3709813049583744	0.6652653753279992
0.6003486717120881	0.3709813049583744	0.1652653902440322
0.6003486716930526	0.3709813049583744	0.6652653753279992
0.9925842201733338	0.0786888195691996	0.1027897791783762
0.9925842201701648	0.0786888195691996	0.6027897717473993
0.4925842201733339	0.0786888195691996	0.1027897791783762
0.4925842201701649	0.0786888195691996	0.6027897717473993
0.9921002996190814	0.4032141422158504	0.1042736543715065
0.9921002996300593	0.4032141422158504	0.6042736543823208
0.4921003294347762	0.4032141422158504	0.1042736543715065
0.4921003294457541	0.4032141422158504	0.6042736543823208

3.3. TATB unit cell

1.0

9.2074735902134108	-0.0063117371180602	0.0004979609244119
-4.6143324124210778	7.9967437309330602	0.0026757353576860
-0.2187825302030979	-2.6869832341083448	6.6372176643706045

H C N O

12 12 12 12

Direct

0.4840524258188916	0.8656187104930450	0.2456814368226092
0.5159475443738334	0.1343812895878150	0.7543185482556803
0.2554058384200267	0.7518386725379259	0.2370781197769617
0.7445941616160620	0.2481613274653633	0.7629218802708081
0.9683394410738937	0.0616517532164466	0.2633064308891876
0.0316605850236379	0.9383482616998700	0.7366935393788555
0.0822198422620079	0.2858352671964248	0.2577150958632777
0.9177801502345896	0.7141647327062808	0.7422848742455177
0.6749854403111035	0.5725253548439552	0.2411660359241372
0.3250145596856775	0.4274746451866128	0.7588339341846587
0.7883774924843145	0.4620588250284631	0.2537659247620234
0.2116225074734935	0.5379411452296325	0.7462341050654885
0.5325657098260963	0.1674195455208862	0.2572571952422169
0.4674342901273638	0.8325804394687848	0.7427428046463060
0.3735142896159832	0.0079781913968918	0.2500710337782819
0.6264857402217385	0.9920218375002562	0.7499289810797348
0.2177942035036919	0.0109885349217718	0.2496888450841576
0.7822057965202247	0.9890114623963838	0.7503111549636123
0.2181631217231541	0.1684645235387216	0.2511925693561637
0.7818368932372737	0.8315354914245977	0.7488074306916133
0.3762043582407840	0.3218571692690597	0.2450336100363366
0.6237956417559978	0.6781428307615082	0.7549663600724592
0.5346861572283067	0.3237216479275596	0.2500210937604952
0.4653138426967952	0.6762783520757302	0.7499789062872746
0.6877444479392975	0.1687693331136406	0.2654683217201210
0.3122555521367520	0.8312306966818460	0.7345316783276487
0.3707829640381940	0.8615270425803939	0.2430631628851023
0.6292170358958958	0.1384729722517668	0.7569368519729144
0.0622849430508421	0.8568515856113101	0.2482854743142288
0.9377150382963026	0.1431484142608280	0.7517145405437883
0.0767910883150775	0.1725140364003689	0.2582560618336155
0.9232088966933589	0.8274859485423073	0.7417439082751809
0.3762070316715527	0.4739929869756465	0.2379705942602219
0.6237929981099085	0.5260070428471257	0.7620293758485739
0.6789355163739065	0.4654282992973092	0.2479301430909784
0.3210644835895219	0.5345717006589784	0.7520698271770645
0.8284753937730256	0.3058851923231869	0.2731592488021482
0.1715246062231089	0.6941147778879060	0.7268407512456280
0.6892277364260574	0.0317764400336200	0.2643528523452971
0.3107722636094838	0.9682235618406210	0.7356471179227455
0.0571407775714192	0.7163472828210523	0.2429971046500264
0.9428592224856801	0.2836527171789480	0.7570029102079907
0.9246593799666484	0.8564631077678622	0.2519515489095049
0.0753406200694400	0.1435368922354279	0.7480484511382647
0.2402987530361312	0.4769141112880320	0.2334964776072617
0.7597012618099559	0.5230859184265958	0.7665035522202427
0.5129658546678986	0.6121240346045794	0.2374921265374747
0.4870341453681900	0.3878759653987109	0.7625078735103019

3.4. TATB (001) surface

```
1.0
   18.4330005646000004    0.0000000000000000    0.0000000000000000
    0.0501486149000000   15.9715208487999991    0.0000000000000000
    0.0000000000000000    0.0000000000000000   20.1763992310000013
   H    C    N    O
   96    96    96    96
Direct
  0.0703373324514936  0.3116238571573147 -0.0138296403982404
  0.5702209003181729  0.3117373917605104 -0.0137812636313731
  0.3202846736817134  0.8116521733155504 -0.0138044199030058
  0.8202676249586783  0.8115915751113257 -0.0140267995061097
  0.1845558640698134  0.7452437011535660  0.3404945853822176
  0.6845578086815078  0.7453300644043677  0.3400406033867735
  0.4348525665533523  0.2452698491168761  0.3403918992144253
  0.9346725376206076  0.2452358557491051  0.3403628200422801
  0.0778356873017545  0.3999027775897674  0.1626471423426443
  0.5778469491602308  0.3998538433618020  0.1629988129923534
  0.3279008864247066  0.8998602634000459  0.1627560359633815
  0.8276179464205861  0.8999134854802768  0.1625995469741369
  0.1946253005166131  0.8307558988177806  0.5195000031042687
  0.6954921800530965  0.8295919944980905  0.5197974985837598
  0.4448117132968167  0.3304014946883733  0.5192055067008108
  0.9449359979367138  0.3306261133561840  0.5190998560558598
  0.9861332394796510  0.3686726716053431 -0.0218243184453674
  0.4860439191873189  0.3687682298015262 -0.0218314209253493
  0.2360880551498094  0.8686627649279706 -0.0217758991871318
  0.7360786618275281  0.8686701784478252 -0.0218840999319133
  0.0999023162417609  0.8026241464760668  0.3377982974973942
  0.5999342221474145  0.8026872883091760  0.3372952154576190
  0.3501465464920713  0.3025368599340426  0.3378429231969734
  0.8500250887854032  0.3026034337660430  0.3377221951975730
  0.1625070534050840  0.3427253754477102  0.1663345037038444
  0.6625515087049703  0.3426539637467718  0.1664819116148009
  0.4125658670583049  0.8426075930583279  0.1663507749301699
  0.9123804407758784  0.8428035547292040  0.1660167726836204
  0.2788874812243644  0.7737447896806444  0.5266103383652623
  0.7799267933172807  0.7726468820229710  0.5264552739416829
  0.5291678143728897  0.2733840683302078  0.5260990138439529
  0.0291920456666352  0.2735540084965548  0.5263817637186513
  0.9862716625239272  0.6659935061048189 -0.0103961254908082
  0.4863119899335259  0.6662067922031529 -0.0101704159944491
  0.2362718039474254  0.1660521939710455 -0.0102119941594062
  0.7362706433880113  0.1659978023383922 -0.0101869409603790
  0.1010716110583856  0.0995960932954985  0.3431549208678399
  0.6010006743330462  0.0995994344145076  0.3434157234859451
  0.3511292838140198  0.5995668469338193  0.3430679410256448
  0.8511027127444992  0.5995275767719301  0.3427313563327535
  0.1620043834760804  0.0458905927431359  0.1614663337649609
  0.6620019060811870  0.0458517256102360  0.1614522541346896
```

0.4119974576384949	0.5458242867315478	0.1615337990022220
0.9120546893469222	0.5459477425646401	0.1611815748176966
0.2785048375805242	0.4757551705559717	0.5155332133569683
0.7791305935401156	0.4748645007224632	0.5146318981424325
0.5287632720693586	0.9756802685051306	0.5148788181672898
0.0287263332797636	0.9757597116591562	0.5149082250263451
0.0702335294763668	0.7222892752711801	-0.0179282945390062
0.5703019678000382	0.7224090633173128	-0.0178996967743060
0.3202413764967501	0.2223407268532615	-0.0178549299988203
0.8203068230618564	0.2222417261651172	-0.0177635459618702
0.1861641032957835	0.1554434957462929	0.3447432699621183
0.6860369375815938	0.1554760346008937	0.3452139567615557
0.4361922761594264	0.6554395789503442	0.3444126558774995
0.9361830862452948	0.6554410114058206	0.3443228521293866
0.0770922410129810	0.9898337837298145	0.1597961139596410
0.5770559537507352	0.9897841177928443	0.1598247866691891
0.3270499535173164	0.4897954779559029	0.1600052191724826
0.8271565383682102	0.4898041657429658	0.1596379284753396
0.1942516335986068	0.4201745679452497	0.5239125846784776
0.6950978197139920	0.4190265564187254	0.5226150436281611
0.4446921400971441	0.9197224223623849	0.5229667754031029
0.9445861884974928	0.9199957411764900	0.5231955875176975
0.2944925130123184	0.5719631577160614	-0.0141789293153500
0.7944504069081638	0.5719527442832012	-0.0144271819153690
0.5444728989413712	0.0719611401839820	-0.0142372921803877
0.0444966424410083	0.0718335044158502	-0.0142264652846958
0.4086656344952197	0.0051821195290973	0.3366775679137886
0.9085383527602645	0.0054301360013586	0.3367195956709111
0.6588235785769391	0.5052135986952349	0.3366896721163581
0.1587222173343139	0.5052442980109231	0.3370037984490312
0.8541424582735841	0.1395721377476641	0.1662677850878198
0.3541180683049786	0.1396068462146774	0.1663422282019428
0.1041676052969715	0.6395699407236862	0.1659836435380830
0.6041827286223784	0.6394602005718573	0.1659450659012287
0.9707114647061637	0.5701431704964038	0.5184918295206229
0.4713930901069934	0.5697353875140325	0.5179133493383152
0.2207816541881514	0.0701728852529440	0.5184068503362117
0.7207523440534275	0.0703508761186949	0.5186747617663063
0.2941199929386992	0.4593335763637172	-0.0191951231212478
0.7940429212155266	0.4593185764861140	-0.0194085595534586
0.5440612519485289	0.9593187039238537	-0.0192022531969228
0.0440848279995967	0.9592087058026978	-0.0193272613229153
0.4083747871615097	0.8921530221460641	0.3380215326208909
0.9084724444944348	0.8924646514894599	0.3380157044854650
0.6585831768504750	0.3922511884052648	0.3384738234588868
0.1585107529506951	0.3922452241814078	0.3384352420176306
0.8542450073186497	0.2525846847233931	0.1646282320568181
0.3542473406942033	0.2526321802383620	0.1646591150083166
0.1042365087660082	0.7525749179732314	0.1642683753836162
0.6041717132581785	0.7524365817957185	0.1641673599113846
0.9705794163193872	0.6825277429365872	0.5246458701721598
0.4714459796693597	0.6821921793058543	0.5241107174281672

0.2209849156696670	0.1826155034122201	0.5249414295179162
0.7210334440251253	0.1828555362235271	0.5248011788540797
0.1559580826346804	0.4384409605293967	-0.0146983812107662
0.6558841878122874	0.4384848204581842	-0.0147625876176926
0.4059119866926122	0.9384538283654645	-0.0147180369494881
0.9059287650234029	0.9383592869685472	-0.0148347919028244
0.2702703585051296	0.8716356427471492	0.3407982794740841
0.7702678070970426	0.8717236684492908	0.3406767250143800
0.5204578680851843	0.3716414695974488	0.3410745017829863
0.0203758193008477	0.3716194712337610	0.3407958177118755
0.9923517603087283	0.2733685686383751	0.1621123453769881
0.4923392950405895	0.2733587103182127	0.1622553663692519
0.2423577793537306	0.7733681737123622	0.1621305194933349
0.7422344371230986	0.7733535751744794	0.1619789970497513
0.1089263981183113	0.7040435824254981	0.5206447476572651
0.6099313063885969	0.7031587556488710	0.5205601298330544
0.3592090378778230	0.2037664784306934	0.5208439486871971
0.8592592061363145	0.2038897136561794	0.5204054769459124
0.0773930624321820	0.4378984418487085	-0.0156754501227698
0.5773101694089197	0.4380083753706818	-0.0156914678236485
0.3273563104723645	0.9379461279257234	-0.0156571580471403
0.8273559638636030	0.9378756871240927	-0.0157416141633620
0.1915261944215992	0.8714007373369282	0.3397841175621639
0.6915332972438434	0.8714809969299839	0.3395487165347374
0.4417261554423735	0.3714271282893366	0.3398754638901292
0.9416165878153909	0.3713921753070626	0.3397451866676226
0.0710510769668482	0.2737062928352288	0.1635581200686495
0.5710468567466730	0.2736915762328004	0.1637352799738982
0.3210664158821310	0.7736774295563533	0.1635988403875906
0.8209186276722028	0.7737458652761791	0.1633699696583315
0.1875695689617952	0.7045268521469179	0.5212100114680014
0.6885306215048629	0.7034830195892022	0.5211646434298162
0.4378297221452550	0.2042077886603230	0.5212701558696750
0.9378587164285221	0.2043701753738507	0.5210192738097867
0.0388995810140433	0.5170547199865297	-0.0153582875819006
0.5388610081440228	0.5171659072399825	-0.0153525991907689
0.2888903782882745	0.0171059886494604	-0.0153095185389742
0.7888678295568862	0.0170206747228798	-0.0152802220326758
0.1531982721608002	0.9506616856276464	0.3392231527265432
0.6531797344199584	0.9507204321854014	0.3390936011267289
0.4033356614036643	0.4506351076281588	0.3392954369997191
0.9032644549567024	0.4506409158800881	0.3391821157372067
0.1095448886714631	0.1946214544520724	0.1648900046596864
0.6095569608666832	0.1945931241703513	0.1649570505734265
0.3595664431033441	0.6946055955531570	0.1648734193548620
0.8594734386721683	0.6946954392200931	0.1644434635066841
0.2261193811442670	0.6253860173636021	0.5205335191810946
0.7269380094120983	0.6242059802874866	0.5201041413694140
0.4763080551196497	0.1250063804284053	0.5204741007120298
0.9763403913058515	0.1252450171858984	0.5203574464447268
0.0775013253710854	0.5958527782187515	-0.0149961171930008
0.5774954463983056	0.5959611491866531	-0.0150290213429900

0.3274932164854676	0.0958970326198582	-0.0149683881107232
0.8274723497284471	0.0958023024732581	-0.0149202622656256
0.1921485808832274	0.0293427881090822	0.3407121881636431
0.6920929338955957	0.0293975777052069	0.3407717032209650
0.4422310584973612	0.5293248420928713	0.3406629421509848
0.9421899294423033	0.5293355640090049	0.3405698535262033
0.0707729888480494	0.1159263191084049	0.1632671251776613
0.5707735303826627	0.1158871008316578	0.1632883730132642
0.3207885782193111	0.6159098034215724	0.1633221739140253
0.8207894607023875	0.6159144446784618	0.1630327348395878
0.1875542592579765	0.5465451254831903	0.5204683406431105
0.6881474134712589	0.5454691819247470	0.5199778889585986
0.4377019929889837	0.0461543666199545	0.5201865415412811
0.9377455967899432	0.0464162483523859	0.5201351637899719
0.1561349723994836	0.5946143356396760	-0.0160690890495963
0.6561286393459390	0.5946781174337948	-0.0161811659077205
0.4061257124333911	0.0946441062996117	-0.0160770060213793
0.9061124904981875	0.0945370956118318	-0.0160029275648981
0.2708333890800719	0.0279976247453221	0.3396187246792836
0.7707789156541968	0.0280727723510075	0.3396181969258270
0.5209229163726585	0.5279997432383070	0.3395419627420133
0.0208970589976949	0.5279758051136382	0.3396133361448656
0.9920928400846691	0.1170819959478822	0.1638068069388103
0.4920898908200915	0.1170851997689351	0.1638063052621213
0.2421073122882802	0.6170830185119094	0.1638694435230079
0.7421055566704561	0.6170172970535501	0.1637430965248217
0.1089069067095609	0.5478966792662632	0.5213028042891537
0.6094927221096027	0.5470247449059179	0.5208475568697869
0.3590636917920526	0.0476218624887669	0.5212008062886951
0.8591072461834235	0.0477906871704530	0.5212011474516310
0.1961186138288200	0.5162573794558030	-0.0157421599105795
0.6960554513590776	0.5162781675820409	-0.0158769161212132
0.4460737922664839	0.0162772861710443	-0.0157602043373049
0.9460940264299997	0.0161751599254833	-0.0157763486535418
0.3105252186044931	0.9495291340229528	0.3391491081864784
0.8104957442860996	0.9496208923040217	0.3390723080234774
0.5606733975173770	0.4495639265880398	0.3392687870075914
0.0606218824517709	0.4495063761314480	0.3392507416521711
0.9521712172869252	0.1954416790285065	0.1638092327163656
0.4521661421837909	0.1954415923317477	0.1638467560145234
0.2021776496248079	0.6954613810835886	0.1637542518155054
0.7021663967192692	0.6953796942054890	0.1636667236752568
0.0689146951378531	0.6262136634503639	0.5211571177965663
0.5696849913848903	0.6254611915172942	0.5207939227250188
0.3191319285108988	0.1259843141316621	0.5212557510075254
0.8191616729430728	0.1261108742153609	0.5211372917008575
0.1944891199588767	0.3609466239141701	-0.0139407879626143
0.6943698707387344	0.3609714309428205	-0.0139612231414058
0.4444356172131839	0.8609630828350995	-0.0139065385392363
0.9444307426739883	0.8608566231929254	-0.0141826180006095
0.3086210004893846	0.7943039076292214	0.3428650971075259
0.8086293977726351	0.7943907440995568	0.3427575798848576

0.5588098551890749	0.2943329490424385	0.3434481754987516
0.0587461478844611	0.2943086698664770	0.3429026993178044
0.9538754956652146	0.3506777274572502	0.1600276931686725
0.4538942658840308	0.3506867934679395	0.1601901978798289
0.2038978014687516	0.8506721705209469	0.1600801277551555
0.7037138800314716	0.8506143381499697	0.1599314169069042
0.0702833813415903	0.7813846496416488	0.5199907012587653
0.5714762264676115	0.7806666523707239	0.5199808205026835
0.3205922139153023	0.2811874813817766	0.5203448335553785
0.8207068050646984	0.2813279154030852	0.5196373273826870
0.0413782241674063	0.3662064086355454	-0.0170812909138931
0.5412751804409757	0.3663445417001855	-0.0170577290910035
0.2913459026279275	0.8662595178346296	-0.0170596240885048
0.7913188325336769	0.8662043645871839	-0.0171799410491925
0.1554082526731615	0.7998446886695136	0.3387467509030789
0.6554391179834889	0.7999297710611475	0.3382655480095199
0.4056565219645201	0.2998427358342095	0.3387091220742863
0.9055244980890176	0.2998214949088501	0.3386298214356385
0.1070268377599233	0.3453635492236877	0.1645509385326711
0.6070592573131411	0.3453175621980216	0.1647810810140833
0.3570796606748918	0.8453047922893850	0.1645794576490931
0.8568764027651694	0.8454142290784709	0.1644692241782387
0.2235650608879148	0.7761889317849413	0.5226992637748600
0.7245935643283397	0.7750927958653842	0.5228731637609138
0.4738268466479061	0.2758563922630070	0.5224590021441478
0.9738677781671820	0.2760169368705588	0.5224479806508862
0.9616302374164750	0.5174000618904609	-0.0159545850545799
0.4615948211570362	0.5175408395288098	-0.0158852468682919
0.2116165766344541	0.0174432723686293	-0.0158324713094247
0.7116030445862439	0.0173973941939533	-0.0158001904528734
0.0759261345718902	0.9512521605854516	0.3382113580489807
0.5759046506014678	0.9512805788405085	0.3382096393538349
0.3260726693891341	0.4511289868191838	0.3383848038178932
0.8260056673617658	0.4512106748120086	0.3382888499494339
0.1868060169450593	0.1943331612628525	0.1671560032291620
0.6868187095269495	0.1942586468704585	0.1672227587837569
0.4368360184191168	0.6942534939306889	0.1671273285177118
0.9367631625015922	0.6944437504901831	0.1662347027581123
0.3033736913160147	0.6249915903073185	0.5200710116925391
0.8041687826029041	0.6236666152525947	0.5195315040705343
0.5535533095594120	0.1246868987078795	0.5200021889358872
0.0536052497218263	0.1248593586002575	0.5200660443153586
0.0415754776986879	0.6677823266608918	-0.0142513659288819
0.5416146926926032	0.6679225211133305	-0.0141286190521329
0.2915809249971792	0.1678285322690776	-0.0141205767793189
0.7915891348930784	0.1677680798635216	-0.0140451594366232
0.1565740338556788	0.1013477651912876	0.3430918189798983
0.6564959840784771	0.1013732211132210	0.3433144184958649
0.4066354401180162	0.6013190951287801	0.3429709800750005
0.9066078863162136	0.6013441384966877	0.3426841671645700
0.1065221086421670	0.0440123723420784	0.1612556923383716
0.6065046997783763	0.0439551822843726	0.1613367638057714

0.3565156837592283	0.5439719259212900	0.1613676577225912
0.8565601185887602	0.5440297978042664	0.1610821707459479
0.2232468730858652	0.4744468620810912	0.5197840335727262
0.7238588941471141	0.4734337531206071	0.5188887381339591
0.4735039271766012	0.9741117758539275	0.5191835156645908
0.9734569969963838	0.9743539759332508	0.5191441762230148
0.1946710008362322	0.6716364554884234	-0.0170572771576518
0.6946926366857177	0.6716717737939403	-0.0171298741069908
0.4446725434445154	0.1716514042967344	-0.0170628253914965
0.9446832467019212	0.1715650373647574	-0.0169956974480019
0.3096009080110845	0.1048174152979211	0.3399310400068847
0.8095157231715979	0.1049105190051069	0.3402024664404337
0.5596496218350586	0.6048642575578368	0.3396946075838373
0.0596506215791926	0.6048113625699097	0.3399592560910560
0.9534750433103650	0.0401895142183909	0.1639441618723081
0.4534356964727488	0.0401864685420611	0.1638906359284268
0.2034654093003309	0.5401743421156102	0.1640809507324657
0.7034969668229053	0.5401117203014651	0.1638131973189218
0.0703586381776589	0.4708232705938413	0.5218934687746503
0.5707927088264542	0.4700766581438338	0.5211911170659623
0.3204669089558793	0.9705825788311081	0.5214395605499272
0.8204942933894963	0.9707470950760666	0.5217566128173917
0.2680010931833137	0.5158663759619595	-0.0165156070578540
0.7679387156849807	0.5158646186360206	-0.0167927465164111
0.5179525075365942	0.0158611939650752	-0.0165859490511041
0.0179820858660834	0.0157567125878750	-0.0166232181156750
0.3823387408351108	0.9488625387714625	0.3380629899706840
0.8823078197779303	0.9490638223239621	0.3380097299904791
0.6324836024780005	0.4489181547612889	0.3381503493313728
0.1324352658364960	0.4489119962412029	0.3383952319177272
0.8803553569266468	0.1959283139971739	0.1646322986516453
0.3803448402899212	0.1959595368858093	0.1646607139247182
0.1303684845132052	0.6959429633333025	0.1643866139284338
0.6303577447411619	0.6958344399148569	0.1642814185885671
0.9970314212400437	0.6262830567280436	0.5217299143871452
0.4977715203630321	0.6258300741510846	0.5211503452247946
0.2472257739038282	0.1262085947444580	0.5218418112609134
0.7472565914930023	0.1263870117694098	0.5219133216243037
0.2623239959939392	0.3586973613563093	-0.0225624298764128
0.7622001981564798	0.3586971149690182	-0.0225996687354661
0.5122823604173021	0.8586929982891687	-0.0224828406707358
0.0122770509148729	0.8586039713055927	-0.0227217425396049
0.3771440596913996	0.7920898783272390	0.3410017037142433
0.8771503018473925	0.7922095039942814	0.3409166531450087
0.6273573862580475	0.2921679902447282	0.3423230676129939
0.1272708701521427	0.2921427243071428	0.3411793859062507
0.8853412655013653	0.3527674175905413	0.1616000340900954
0.3853493190962273	0.3528077819957143	0.1615601998289723
0.1353548589900722	0.8527710229600177	0.1616039463045372
0.6351818345676324	0.8526595406816965	0.1615322617788251
0.0024505105471463	0.7834140283623907	0.5286741795448148
0.5036454028253824	0.7828346394197002	0.5284690826959262

0.2528105392550952	0.2832819754841465	0.5293622454540693
0.7528881677068556	0.2835018094782723	0.5284097516508233
0.1615413969018430	0.2926104317533922	-0.0045733636880160
0.6613833257980299	0.2926785723478078	-0.0044877814035769
0.4114856124022135	0.7926273507324315	-0.0045174736835833
0.9114407579088150	0.7925170956210765	-0.0049358369309218
0.2751271950617960	0.7252264996123835	0.3464783055610562
0.7751308317939585	0.7253377613928352	0.3463733627588014
0.5253168893233434	0.2252204638577253	0.3467108220956103
0.0252508196403512	0.2252195446543931	0.3464016584395174
0.9872943440794000	0.4198346046415216	0.1566910234396638
0.4873404906909533	0.4198317508254789	0.1571012778037525
0.2373489754117581	0.9198049209724168	0.1568665782554671
0.7371130985592886	0.9197927786525553	0.1566979392918490
0.1032037499944341	0.8497369086559993	0.5106757444172639
0.6045112850050820	0.8490059476830945	0.5109348324254284
0.3534632174065261	0.3495308985651948	0.5108427433894716
0.8535792390744044	0.3496552433802776	0.5100393794591601
0.9265204744501095	0.4507898511625144	-0.0261608840671023
0.4264585253200280	0.4510010102876224	-0.0262317002224820
0.1764922742037122	0.9508608771684646	-0.0261084428903728
0.6764626929044748	0.9508181433825843	-0.0260808261898800
0.0403678473811285	0.8838005010480096	0.3386558822260096
0.5403676366059388	0.8838057992523262	0.3387176342061924
0.2905719556792795	0.3836466386293113	0.3391307789384184
0.7904599567813619	0.3837544309729654	0.3389101787631544
0.2222055535632877	0.2619022756882280	0.1674980811984890
0.7222442706273835	0.2618110303327077	0.1676925181546064
0.4722617861551595	0.7617967877453450	0.1675801168683813
0.9721445944198188	0.7620333673154764	0.1660782914875187
0.3386573100867203	0.6916021358708699	0.5297035226095047
0.8395435539195575	0.6903614223156138	0.5286003440514144
0.5887709690932210	0.1913671164968822	0.5295742149235613
0.0888270442183269	0.1914787769375184	0.5298913564365785
0.9263829609129447	0.5843566665949538	-0.0064126010585423
0.4264034712902452	0.5845052096970677	-0.0061566366294063
0.1763956566320856	0.0843855697755347	-0.0061632415079466
0.6763873459309752	0.0843660941629932	-0.0061734843630602
0.0408349510153538	0.0194892153129076	0.3366525737642293
0.5407881232354937	0.0195069343190846	0.3367121761558369
0.2909379366343072	0.5193429846904922	0.3366622833524732
0.7909168737571965	0.5194479046718544	0.3366843225529859
0.2220278233880495	0.1262089920949614	0.1691648240912956
0.7220466691298678	0.1261151042636106	0.1690974268780768
0.4720398305368561	0.6261170605901876	0.1690399659080172
0.9720219314664407	0.6263531588630888	0.1682822888748395
0.3384090922376913	0.5579948665200851	0.5101066487895501
0.8392121849141305	0.5565067773535017	0.5100974442720139
0.5886794738090864	0.0577003191892892	0.5101514055681367
0.0886880747064241	0.0578716107924454	0.5101233981461625
0.1619071062702583	0.7403698938172784	-0.0258981495120197
0.6619869978553589	0.7404305728244976	-0.0259947895518201

0.4119373754044151	0.2403976826186120	-0.0258723573306726
0.9119449729746930	0.2403358254121215	-0.0257731584211175
0.2767875473451487	0.1743861012708466	0.3428820719792974
0.7766669700323947	0.1744431576122966	0.3434558454435833
0.5267814718027309	0.6744296267526055	0.3421167832082954
0.0268183631631010	0.6743766944656557	0.3427876795067005
0.9864688870872281	0.9706674231108182	0.1621532072728540
0.4863860278229972	0.9706485879897587	0.1620412597527737
0.2364177273542846	0.4706274268588962	0.1624664995678853
0.7365021781728993	0.4706037477668550	0.1619766429180080
0.1027930765340559	0.4022187361752853	0.5321915643786116
0.6031738846667445	0.4013383874300943	0.5310041196105851
0.3529513109900315	0.9018963241101747	0.5312565128246626
0.8529666400633055	0.9021190936227922	0.5318705925675248
0.2627771979425885	0.6731542363401992	-0.0087840425874237
0.7627965736997436	0.6731208097333727	-0.0088294639784359
0.5127842611907888	0.1731420466012708	-0.0088434737565554
0.0127876070149944	0.1730534449850266	-0.0087814290077555
0.3782958909099849	0.1058187345837748	0.3376962022993317
0.8782255324537113	0.1059696473282810	0.3379237841174411
0.6283621765311800	0.6058732680302881	0.3378387928170098
0.1283602995572685	0.6058440872954427	0.3378268603288362
0.8847397726245509	0.0390698740631746	0.1653898952391507
0.3846977990857607	0.0390924956631881	0.1653966892977681
0.1347135915855873	0.5390473512779429	0.1654964812244054
0.6347601051492671	0.5389694284971808	0.1653133188894234
0.0025569103080143	0.4691086140407375	0.5118025448873613
0.5029347473668181	0.4686728976152121	0.5113537411503760
0.2526407891821131	0.9690338099844258	0.5115393411476883
0.7526736767822393	0.9691624938569715	0.5118374889191663

References

1. Cady, H.H.; Larson, A.C. The crystal structure of 1,3,5-triamino-2,4,6-trinitrobenzene. *Acta Crystallogr.* **1965**, *18*, 458–496.
